# The evolutionary conserved TLDc domain defines a new class of (H^+^)V-ATPase interacting proteins

**DOI:** 10.1038/s41598-021-01809-y

**Published:** 2021-11-22

**Authors:** A. F. Eaton, D. Brown, M. Merkulova

**Affiliations:** 1 Program in Membrane Biology and Division of Nephrology, Massachusetts General Hospital and Harvard Medical School, Boston, MA 02114 USA; 2 Program in Membrane Biology and Division of Nephrology, Massachusetts General Hospital, Simches Research Center, 128 Cambridge St., Boston, MA 02114 USA

**Keywords:** Biochemistry, Ion pumps

## Abstract

We recently found that nuclear receptor coactivator 7 (Ncoa7) and Oxr1 interact with the proton-pumping V-ATPase. Ncoa7 and Oxr1 belong to a group of proteins playing a role in the oxidative stress response, that contain the conserved “TLDc” domain. Here we asked if the three other proteins in this family, i.e., Tbc1d24, Tldc1 and Tldc2 also interact with the V-ATPase and if the TLDc domains are involved in all these interactions. By co-immunoprecipitation, endogenous kidney Tbc1d24 (and Ncoa7 and Oxr1) and overexpressed Tldc1 and Tldc2, all interacted with the V-ATPase. In addition, purified TLDc domains of Ncoa7, Oxr1 and Tldc2 (but not Tbc1d24 or Tldc1) interacted with V-ATPase in GST pull-downs. At the amino acid level, point mutations G815A, G845A and G896A in conserved regions of the Ncoa7 TLDc domain abolished interaction with the V-ATPase, and S817A, L926A and E938A mutations resulted in decreased interaction. Furthermore, poly-E motifs upstream of the TLDc domain in Ncoa7 and Tldc2 show a (nonsignificant) trend towards enhancing the interaction with V-ATPase. Our principal finding is that all five members of the TLDc family of proteins interact with the V-ATPase. We conclude that the TLDc motif defines a new class of V-ATPase interacting regulatory proteins.

## Introduction

The vacuolar ATPase (V-ATPase, also known as the H^+^-ATPase) is a multisubunit, transmembrane protein complex whose function is to catalyze ATP hydrolysis and harness the released energy to actively pump protons across biological membranes. While the V-ATPase is ancient and evolutionarily conserved, it is more complex and diverse in mammals, with multiple isoforms and splice variants existing for several of the V-ATPase subunits^1,2^. There are both ubiquitous and tissue, or cell-type, specific subunit isoforms. For example, the B2 subunit isoform is ubiquitous, while the alternate B1 subunit isoform was originally described as being kidney-specific^3^, but is now known to be expressed in other organs, such as the epididymis, inner ear and olfactory epithelium^1^. V-ATPase complexes containing the more ubiquitous B2 isoform play a critical role in acidification of lysosomes and other specialized intracellular vesicles, such as synaptic vesicles and phagosomes. On the other hand, V-ATPases containing the B1 subunit are usually trafficked to the plasma membrane of specialized cells where they pump protons into the extracellular milieu; for example, during proton transport into the urine, which allows for the maintenance of acid–base homeostasis at the whole-body level^4,5^.

Although the V-ATPase is a housekeeping protein that is indispensable for normal cell functioning and survival, its dysfunctional activity also drives the progression of pathological processes, such as cancer, and neurodegeneration; infection by viruses and other microorganisms also depends on vesicle acidification^1,6–8^. Therefore, the ability to regulate V-ATPase activity with a therapeutic purpose in mind is important, although this must be done in a highly specific manner by targeting pools of V-ATPase that are involved in disease states. To achieve this goal, it is necessary to better understand the pathways that regulate V-ATPase activity, including an improved definition of protein–protein interactions within these pathways.

Our lab recently performed a proteomics analysis of V-ATPase interactions in the kidney, using antibodies to the highly specific B1 subunit isoform of the V-ATPase. We uncovered several previously unknown proteins and protein groups that interacted with the V-ATPase with high probability scores, including Ncoa7 and its homologue, Oxr1^9^. Ncoa7 and Oxr1 are members of a protein family that contain a conserved “TLDc” domain on their C-terminus, and all have been shown to play a role in protecting cells from oxidative stress^10^. These proteins contain two conserved domains: an N-terminal lysin motif (LysM) domain and a C-terminal Tre-2/Bub2/Cdc16 (TBC) and LysM Domain containing (TLDc) domain. One more protein, also containing a TLDc domain, Tbc1d24, was detected in our proteomics study, but did not reach the threshold to be reliably considered as a V-ATPase-interacting protein, possibly due to the high stringency of the protein–protein interaction scoring that we applied in order to avoid possible false positives^9^. In mammals, this family also includes Tldc1 (also known as Meak7) and Tldc2^10,11^. Interestingly, Tldc2 is highly specific to B type kidney intercalated cells^12^. Furthermore, Ncoa7 and Oxr1 genes also give rise to short isoforms known as Ncoa7-B and Oxr1-C^10^. The structure of the TLDc domain is unique and does not resemble that of any other enzymes involved in the inactivation of reactive oxygen species; moreover, it does not appear to exhibit enzymatic activity itself^10,13^. Thus, the molecular mechanisms underlying the antioxidant effects of these proteins, and in particular their TLDc domains, are currently unknown.

As mentioned above, the best-established common biological function of this TLDc group of proteins is protection from oxidative stress. Protective properties were demonstrated for all of these proteins in vitro, and for Oxr1, Tbc1d24 and Ncoa7 in vivo in the central nervous system, which is particularly vulnerable to oxidative stress^10^. It was shown that loss of Oxr1 results in cerebellar neurodegeneration both in mice and humans; thus, Oxr1 plays an essential role in neuroprotection^14,15^. Another TLDc protein, Tbc1d24 also plays an important role in normal brain function. Loss-of-function mutations in this gene in both the TBC and TLDc domains cause epilepsies and other aberrant neurological features in humans and seizures in mice^16,17^. Mechanistically, Tbc1d24 is required for the maturation and oxidative stress resistance of neurons, as well as, synaptic vesicle trafficking in mice^18^. Interestingly, more recently, loss of Ncoa7 in humans was linked to autism spectrum disorders, and although the mechanism remains unknown, it could be due to decreased neuroprotection, similar to Oxr1^19^. It is not known if Tldc1 and Tldc2 play essential roles in protection from oxidative stress in vivo.

Currently, a new role for the TLDc proteins as V-ATPase regulatory proteins is emerging; they are involved in extracellular proton secretion, vesicle acidification and lysosomal function. We showed that Ncoa7 interacts with V-ATPase in the kidney and is highly expressed in proton secreting collecting duct intercalated cells. Furthermore, deletion of Ncoa7 in mice resulted in downregulation of V-ATPase expression and less efficient urine acidification^9,20^, consistent with the function of intercalated cells in regulating systemic acid/base balance via V-ATPase-dependent proton secretion^1^.

Subsequently, in agreement with our results, Ncoa7 and Ncoa7-B were shown to interact with V-ATPase in mouse brain^21^ and in a human glioma cell line where it promotes lysosomal acidification^22^. Loss of Oxr1 resulted in the accumulation of aberrant lysosomal structures, suggesting that it is required for normal lysosomal function^15^. Moreover, Tldc1 localizes to lysosomes, although its interaction with the V-ATPase or its role in lysosomal acidification were not examined in this study^23^. Additionally, Tbc1d24 is required for synaptic vesicle trafficking, suggesting a functional link with V-ATPase in this compartment where the V-ATPase also plays an important role^18,24^. It is noteworthy that the V-ATPase, similar to TLDc proteins also plays a role in protection from oxidative stress^25^, and it is possible that protein complexes formed by TLDc proteins and the V-ATPase are involved in this process.

In this study, we show that all five members of the TLDc family interact with the V-ATPase, and that the TLDc motif itself is critical for this association to occur. Our major finding is that the TLDc motif is a novel protein–protein interaction domain, that defines a new class of V-ATPase-associating proteins.

## Results

### The isolated, purified TLDc domains of Ncoa7, Oxr1 and Tldc2 interact with the V-ATPase in GST pull-down assays

**Figure 1:**
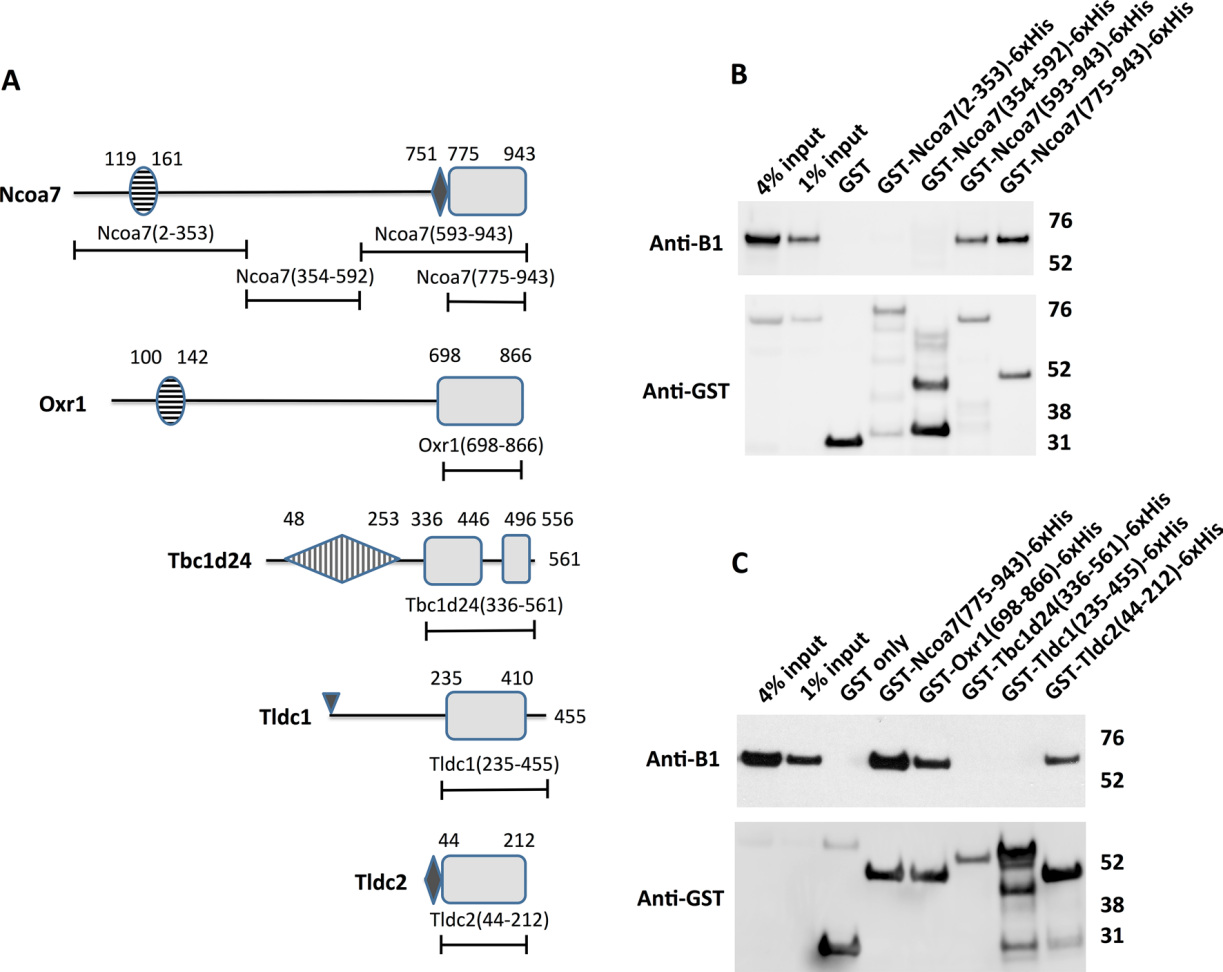
The TLDc domain is sufficient to mediate interaction between TLDc protein family members Ncoa7, Oxr1 and Tldc2 and the kidney-specific B1 subunit of V-ATPase in GST pull-down assay. (**A**) Schematic representation of domain architecture of the TLDc proteins and constructs used to study the role of the conserved TLDc domain in the interaction with V-ATPase. Boundaries of the domains and constructs are indicated as amino acid numbers, as in the longest known isoform of the corresponding mouse protein. The conserved regions of TLDc domains are shown as grey rectangles, the non-conserved loop in the TLDc domain of Tbc1d24 and C-terminal extensions in the TLDc domains of both Tbc1d24 and Tldc1 are shown as lines. The horizontally striped ovals are LysM domains in Ncoa7 and Oxr1. The small black rhombuses in Ncoa7 and Tldc2 are poly-E rich motifs. The vertically striped rhombus in Tbc1d24 is a TBC domain. The small triangle at the beginning of Tldc1 indicates its site of myristylation. (**B**) Anti-B1 and anti-GST western blots of a representative GST pull-down assay using the purified GST-tagged N-terminal region of Ncoa7 (2–353), the middle region of Ncoa7 (354–592), the C-terminal region of Ncoa7 (593–943), and the Ncoa7 TLDc domain (775–943) as baits and kidney lysate containing the endogenous B1 subunit of V-ATPase as a prey. Numbers in parentheses indicate the amino acid boundaries of the constructs, based on of the longest known mouse isoform of Ncoa7. This experiment was repeated five times with similar results. (**C**) Anti-B1 and anti-GST western blots of a representative GST pull-down assay, using the purified GST-tagged TLDc domains of Ncoa7 (775–943), Oxr1 (698–866), Tbc1d24 (336–561), Tldc1 (235–455) and Tldc2 (44–212) and kidney lysate as the source of the B1 subunit of V-ATPase. In both panels (**B**) and (**C**) GST only pull-down was used as a negative control; anti-GST blot was used as a loading control for comparison between samples. This experiment was repeated three times with similar results.

As mentioned in introduction, the interaction between V-ATPase and Ncoa7 was originally identified in immunoprecipitation studies with anti-B1 subunit antibodies. Therefore, we initially focused on details of the interaction between Ncoa7 and the V-ATPase B1 subunit. Mouse Ncoa7 is a large 943 amino acid long protein (Fig. 1A). To study the molecular details of its interaction with V-ATPase, we first narrowed down its interaction site by dividing it into three approximately equal parts: (1) N-terminal Ncoa7 (2–353), which contains a LysM domain of unknown function, (2) middle Ncoa7 (354–592), without known motifs or domains, and (3) C-terminal Ncoa7 (593–943), which contains the TLDc domain and an upstream poly-E motif. The numbering is based on protein sequence NP_766083 of the Ncoa7 long isoform and boundaries of the domains are predicted using NCBI’s Conserved Domains Search tool (CD-Search)^26^. These three regions of mouse Ncoa7, as well as its stand-alone TLDc domain (775–943), were expressed in bacteria with N-terminal GST- and C-terminal 6xHis-tags, purified and used as baits in GST pull-down experiments with mouse kidney protein lysates. Interaction with the V-ATPase B1 subunit was assessed by western blot analysis with anti-B1 subunit antibodies. The C-terminal part of Ncoa7 (593–943) interacted with the B1 subunit, while N-terminal and middle regions did not (Fig. 1B). Then, using the same approach, we further narrowed down the interaction site to the isolated TLDc domain of Ncoa7 (775–943) (Fig. 1B, last lane).

**Figure 2:**
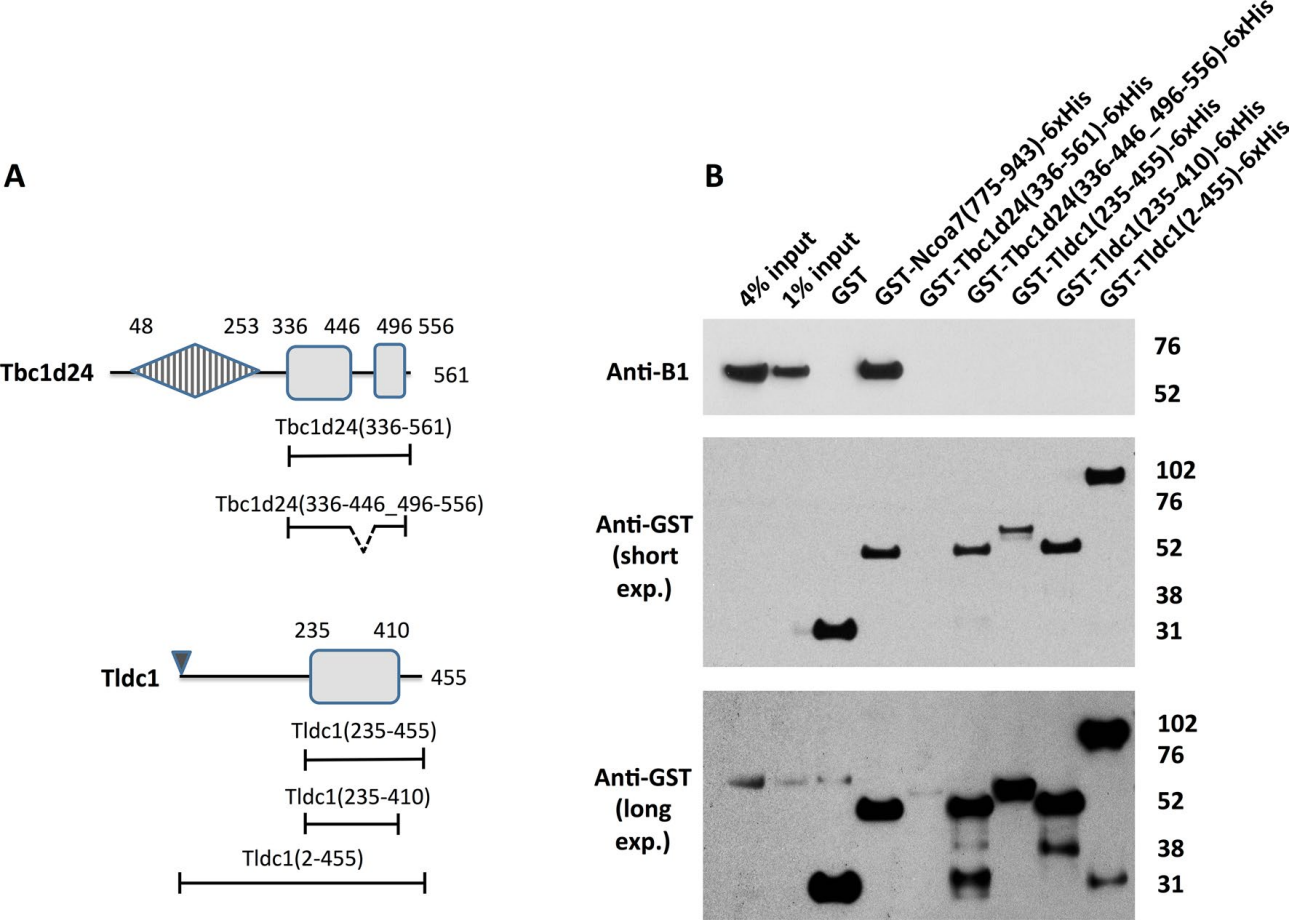
Deletion of a non-conserved loop in the TLDc domain of Tbc1d24 and the C-terminal extensions of both Tbc1d24 and Tldc1, does not result in their interaction with V-ATPase in GST pull down assay. The GST-tagged full-length purified recombinant Tldc1 (2–455) does not interact with V-ATPase in GST pull-down assay. (**A**) Schematic representation of the domain architecture of Tbc1d24 and Tldc1 proteins and the constructs used to study the role of the non-conserved insertion in Tbc1d24 and the C-terminal extensions in both Tbc1d24 and Tldc1 in their interaction with V-ATPase. Boundaries of the domains, non-conserved regions and constructs are indicated as in Fig. 1. (**B**) Anti-B1 and anti-GST western blots of a representative GST pull-down assay, using the purified GST-tagged TLDc domains of Tbc1d24 (336–561) and Tldc1 (235–455), as well as, the GST-tagged truncated versions of the TLDc domains of Tbc1d24 and Tldc1: Tbc1d24 (336-446_496-556) and Tldc1 (235–410), and the GST-tagged full-length recombinant Tldc1 (2–455) with kidney lysate as the source of the B1 subunit of V-ATPase. GST only pull-down was used as a negative control; pull-down with the GST-tagged TLDc domain of Ncoa7 (775–943) was used as a positive control, anti-GST blot was used as a loading control for comparison between samples. This experiment was repeated three times with similar results. Longer exposure of anti-GST blot is shown to confirm the presence of a relatively low amount of GST-tagged Tbc1d24 (336–561) in the pull-down assay.

We then asked if the TLDc domains of Oxr1, Tbc1d24, Tldc1 and Tldc2 also interact with the V-ATPase. To answer this question, we first identified the TLDc domain boundaries in these proteins by performing multiple sequence alignments of mouse Ncoa7, Oxr1, Tbc1d24, Tldc1 and Tldc2. In addition, we examined human Tbc1d24, in which disease-causing mutations are well characterized, and zebrafish Oxr2, whose structure is solved^13^ (Fig. S1). Based on this alignment, the amino acids that correspond to P775 found at the start of the conserved TLDc domain of Ncoa7, are P698 in Oxr1, H336 in Tbc1d24, D235 in Tldc1 and P44 in Tldc2 (numbering is based on the longest isoforms of all proteins, Fig. S1). The conserved regions extend to the very end of Ncoa7, Oxr1 and Tldc2, while Tbc1d24 and Tldc1 contain additional non-conserved amino acid sequences at the C-terminus, and Tbc1d24 contains a long non-conserved loop within its TLDc domain (Fig. S1). In our initial experiments, these non-conserved regions were included in the TLDc domain constructs to better resemble the native proteins. Thus, Oxr1 (698–866), Tbc1d24 (336–561), Tldc1 (235–455) and Tldc2 (44–212), representing TLDc domains of the corresponding proteins, were expressed in bacteria, purified and used in GST pull-down experiments with mouse kidney lysates. We found, that the TLDc domains of Oxr1 and Tldc2, but not Tbc1d24 or Tldc1 interacted with the V-ATPase in these experiments (Fig. 1C). Using the same approach, we also found that even the full-length purified Tldc1 construct (2–455) did not interact with the V-ATPase in GST pull-down experiments (Fig. 2B, last lane). The absence of interaction of Tbc1d24 or Tldc1 and the V-ATPase was unexpected, so we performed additional GST pull-down experiments with truncated versions of the TLDc domains of Tbc1d24 and Tldc1 and also co-immunoprecipitation studies.

### Truncated versions of Tbc1d24 and Tldc1 TLDc domains, lacking the non-conserved insertion and non-conserved C-terminal extensions, respectively, did not interact with V-ATPase

As mentioned above, Tbc1d24 contains a long non-conserved loop (447–495 a.a. in mouse Tbc1d24), in addition both Tbc1d24 and Tldc1 (but not Ncoa7, Oxr1 or Tldc2) have non-conserved C-terminal sequences, extending beyond their TLDc domains (557–561 a.a. in mouse Tbc1d24 and 411–455 a.a. in mouse Tldc1, Fig. 2A). The indicated boundaries are based on the alignment between all 5 TLDc-domain containing mouse proteins (Fig. S1). We hypothesized that these additional regions in Tbc1d24 and Tldc1 might play a regulatory role and prevent interaction with the V-ATPase under our experimental conditions. Therefore, truncated versions of Tbc1d24 and Tldc1, referred to as Tbc1d24 (336-446_496-556) and Tldc1 (235–410), were produced and used as baits in GST pull-downs. However, similar to the non-modified TLDc domains, these truncated versions still did not bind to the V-ATPase (Fig. 2B). These results suggest that the non-conserved extensions in the TLDc domains of Tbc1d24 and Tldc1 are not the explanation for the lack of interaction of these two TLDc domains with the V-ATPase.

### Tbc1d24 protein, but not Tldc1 and Tldc2, is detected in the kidney; endogenous kidney Tbc1d24 and exogenous Tldc1 and Tldc2 overexpressed in HEK293T cells interact with V-ATPase in co-immunoprecipitation studies

**Figure 3:**
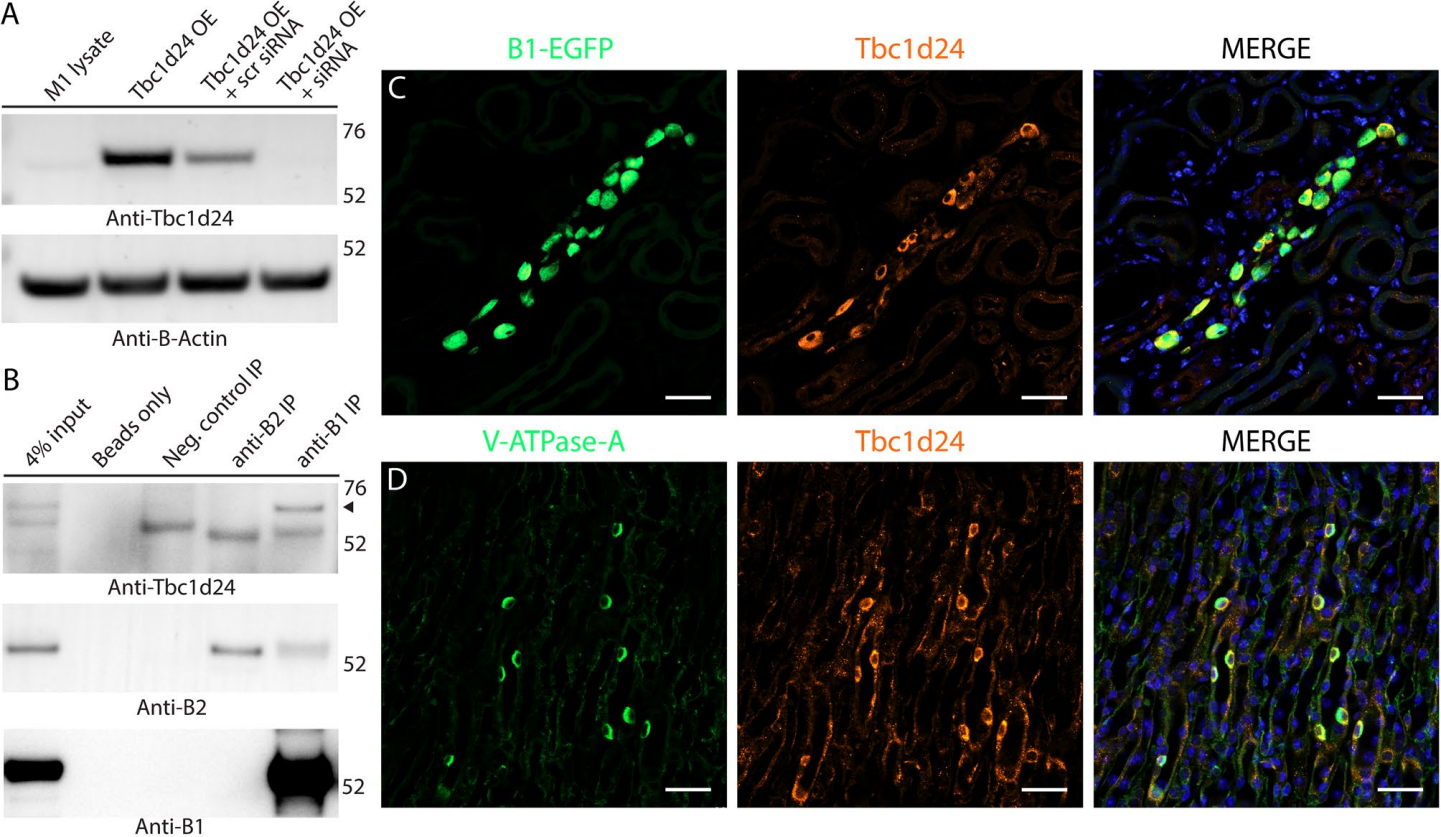
Tbc1d24 is expressed in mouse kidney intercalated cells (ICs) and co-immunoprecipitates with the kidney-specific B1 subunit of V-ATPase. (**A**) Validation of the commercial anti-Tbc1d24 antibodies, used in this study. Anti-Tbc1d24 western blot demonstrates that a band of the expected 63 kDa molecular mass is present in the lysate of M-1 cells overexpressing mouse Tbc1d24 (Tbc1d24 OE), but not in the lysate of M-1 cells co-transfected with both Tbc1d24-expressing plasmids and Tbc1d24-specific siRNA (Tbc1d24 OE + siRNA), confirming specificity of anti-Tbc1d24 antibodies. Anti-β-actin blot was used as a loading control. (**B**) Tbc1d24 co-immunoprecipitates with the kidney-enriched B1 subunit of the V-ATPase, but not with the ubiquitously expressed B2 subunit of V-ATPase in kidney. Proteins were co-immunoprecipitated using anti-B1 and anti-B2 antibodies from mouse kidney lysates and then analyzed by western blot, using anti-Tbc1d24 antibodies. The specific band of the expected 63 kDa molecular mass (arrowhead) is present only in the anti-B1 immunoprecipitation (anti-B1 IP) lane. Note, that the 50 kDa heavy chains of antibodies, used for immunoprecipitation, are detected in anti-B1 IP, anti-B2 IP and isotype control antibodies IP (neg. control IP) lanes, as expected. Anti-B1 and anti-B2 western blots are shown to confirm the successful immunoprecipitation of B1 and B2 subunits of V-ATPase. Anti-B1 antibodies co-immunoprecipitate the B2 subunit of V-ATPase, while anti-B2 antibodies do not co-immunoprecipitate the B1 subunit of V-ATPase, likely because there are many more homo B2/B2 complexes in the whole kidney, than hybrid B1/B2 complexes. This experiment was repeated three times with similar results. (**C**,**D**). Tbc1d24 is co-expressed with V-ATPase in mouse kidney intercalated cells in both cortical and medullary collecting ducts. Immunofluorescence micrographs of cortical (**C**) and inner medullary (**D**) regions of kidney sections from mice expressing EGFP (expressed under promoter of the B1 subunit of V-ATPase) in intercalated cells (**C**, green) or labeled with antibodies against the A subunit of V-ATPase in normal WT mice not expressing EGFP (**D**, green). Intercalated cells identified by their high levels of EGFP expression (**C**, green) or by expression of the A subunit of V-ATPase (**D**, green), and also express high levels of Tbc1d24 (red). In the medulla in particular, Tbcd124 is expressed in some other cell types at lower levels, including collecting duct principal cells. Nuclei are counterstained with DAPI (blue). Scale bar = 20 μm. This experiment was repeated three times with similar results.

In parallel with GST pull-down experiments with the isolated purified TLDc domains, we also studied interaction between the V-ATPase and endogenous full-length Tbc1d24, Tldc1 and Tldc2 by co-immunoprecipitation of these proteins from mouse kidney lysates. Mouse kidney lysate was chosen for these studies initially, because kidneys express high levels of V-ATPase and we found previously that Ncoa7 and Oxr1 are expressed and interact with V-ATPase in kidney^9^. In preliminary experiments the specificity of commercially available antibodies against Tbc1d24 was confirmed by western blot analysis of lysates of mouse cortical collecting duct M-1 cells transfected with a plasmid expressing mouse Tbc1d24 with or without Tbc1d24-specific siRNAs (Fig. 3A). These anti-Tbc1d24 antibodies were able to detect the endogenous Tbc1d24 in the kidney by western blotting (Fig. 3B, input lane) and in kidney intercalated cells in both the cortical (Fig. 3C) and medullary collecting ducts (Fig. 3D) by immunofluorescence. We then performed a co-immunoprecipitation experiment using anti-B1 and anti-B2 antibodies and mouse kidney lysate. Interestingly, Tbc1d24 was detected only in the anti-B1 co-immunoprecipitated material, demonstrating its interaction with V-ATPase and also a preference towards interaction with the B1 subunit of V-ATPase in the kidney (Fig. 3B).

**Figure 4:**
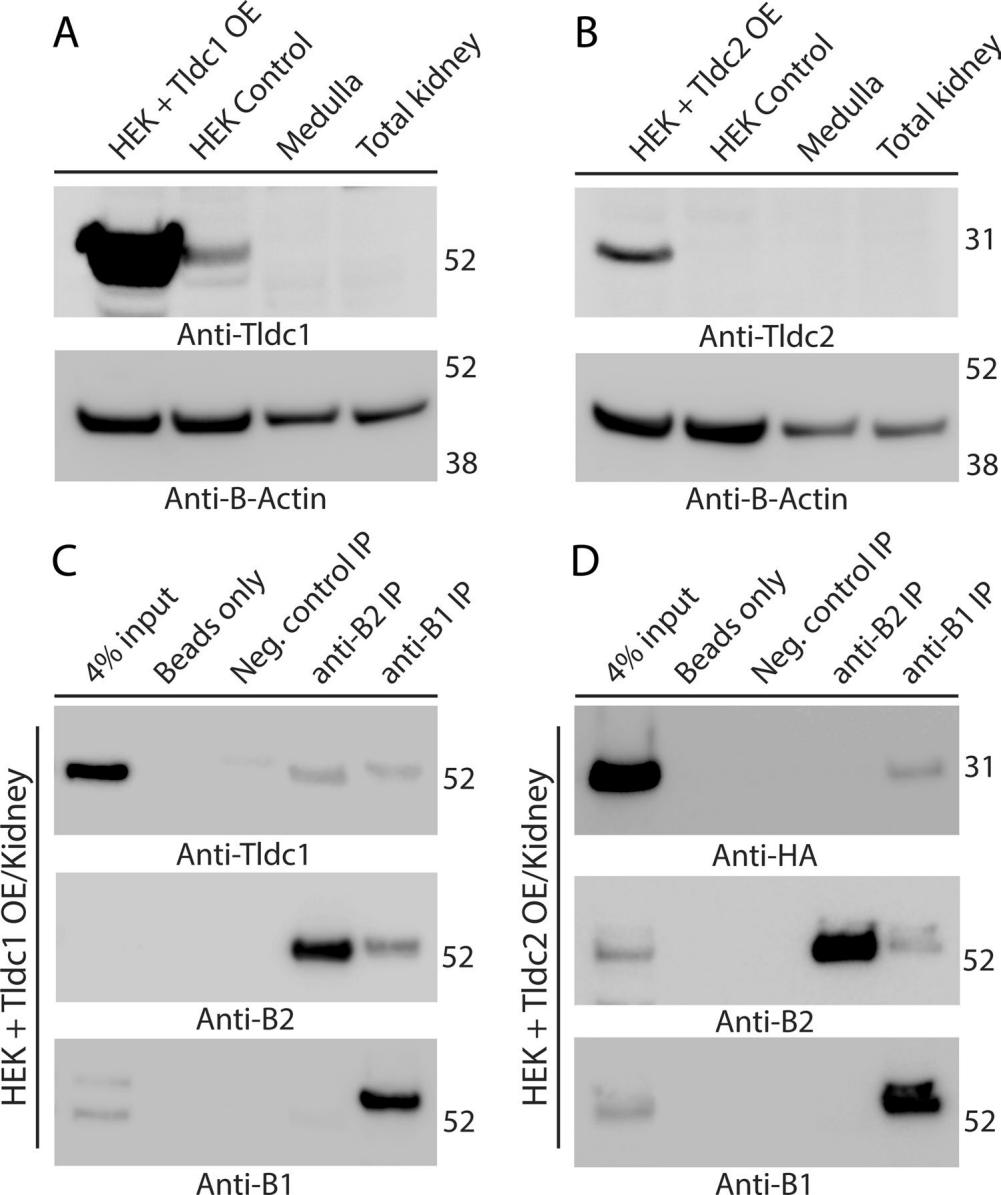
Tldc1 and Tldc2, overexpressed (OE) in HEK293T cells, co-immunoprecipitate with the B1 or B2 subunit of kidney V-ATPase. (**A**) Validation of the commercial anti-Tldc1 antibodies used in this study. Anti-Tldc1 western blot demonstrates that the strong band of the expected 51-kDa molecular mass is present in the lane containing HEK293T lysate from cells overexpressing mouse Tldc1 (HEK + Tldc1 OE), a much weaker band of apparently endogenous Tldc1 is present in the lane containing lysate from untransfected HEK293T cells (HEK control). The expected band of 51-kDa is not detectable in lanes containing total or medullary mouse kidney lysates. (**B**) Validation of the commercial anti-Tldc2 antibodies used in this study. Anti-Tldc2 western blot demonstrates that the strong band of the expected 24-kDa molecular mass is present in the lane containing lysate from HEK293T cells overexpressing mouse Tldc2 (HEK + Tldc2 OE), but the expected band of 24-kDa is not detectable in the lane containing lysate from untransfected HEK293T cells (HEK control), nor in total or medullary mouse kidney lysates. In both panels (**A**) and (**B**) anti-β-actin blot was used as a loading control. (**C**) Tldc1 co-immunoprecipitates with both the B1 and B2 subunit of the V-ATPase. Proteins were co-immunoprecipitated using anti-B1 and anti-B2 antibodies from mixed mouse kidney lysate and HEK293T + Tldc1 overexpressing lysate and then analyzed by western blot, using anti-Tldc1 antibodies. The specific band of the expected 51 kDa molecular mass is present in both the anti-B1 immunoprecipitation (anti-B1 IP) and anti-B2 immunoprecipitation (anti-B2 IP) lanes. This experiment was repeated three times with similar results. (**D**) Tldc2 co-immunoprecipitates with the kidney-enriched B1 subunit of V-ATPase (V-ATPase), but not with the ubiquitously expressed B2 subunit of V-ATPase. Proteins were co-immunoprecipitated using anti-B1 and anti-B2 antibodies from mixed mouse kidney lysate and HEK293T + Tldc2 overexpressing lysate and then analyzed by western blot, using HRP-conjugated anti-HA antibodies, which recognize the HA-tagged overexpressed Tldc2 protein. The specific band of the expected 24 kDa molecular mass is present only in the anti-B1 immunoprecipitation (anti-B1 IP) lane. In both panels (**C**) and (**D**) anti-B1 and anti-B2 western blots are shown to confirm the successful immunoprecipitation of B1 and B2 subunits of V-ATPase. This experiment was repeated three times with similar results.

We also confirmed the specificity of commercial anti-Tldc1 and anti-Tldc2 antibodies, using lysates of HEK293T cells overexpressing mouse Tldc1 (Fig. 4A) or Tldc2 (Fig. 4B). However, these antibodies were not able to detect endogenous levels of Tldc1 (Fig. 4A) or Tldc2 (Fig. 4B) in total or medullary mouse kidney lysates. This could be due to a low level of expression of Tldc1 and Tldc2 in the kidney, or their expression could be limited to a very small number of cells in the kidney. Because proteins can be concentrated during immunoprecipitation and sometimes reach detectable levels, we proceeded with studies using anti-B1 and anti-B2 antibodies for immunoprecipitation, followed by western blotting with anti-Tldc1 and anti-Tldc2 antibodies. However, we were not able to detect Tldc1 or Tldc2 in the co-immunoprecipitated material using kidney lysates (Fig. S2).

Since Tldc1 and Tldc2 were not detected in kidney by western blotting, possibly due to their low expression levels, we overexpressed them in HEK293T cells. We then studied their co-immunoprecipitation with V-ATPase using kidney lysate, mixed with the lysates of HEK293T cells, overexpressing Tldc1 or Tldc2, using anti-B2 and anti-B1 antibodies. In this way, we were able to show that both Tldc1 (Fig. 4C) and Tldc2 (Fig. 4D) interacted with the B1 subunit of V-ATPase. In addition, Tldc1 but not Tldc2 interacted with B2 subunit of V-ATPase (Fig. 4C,D), demonstrating that Tldc2 interacts preferentially with the B1 subunit of V-ATPase.

### A poly-E rich motif, located upstream of the TLDc domain in Ncoa7 and Tldc2, exhibit a (nonsignificant) trend to enhance their interaction with the V-ATPase

**Figure 5:**
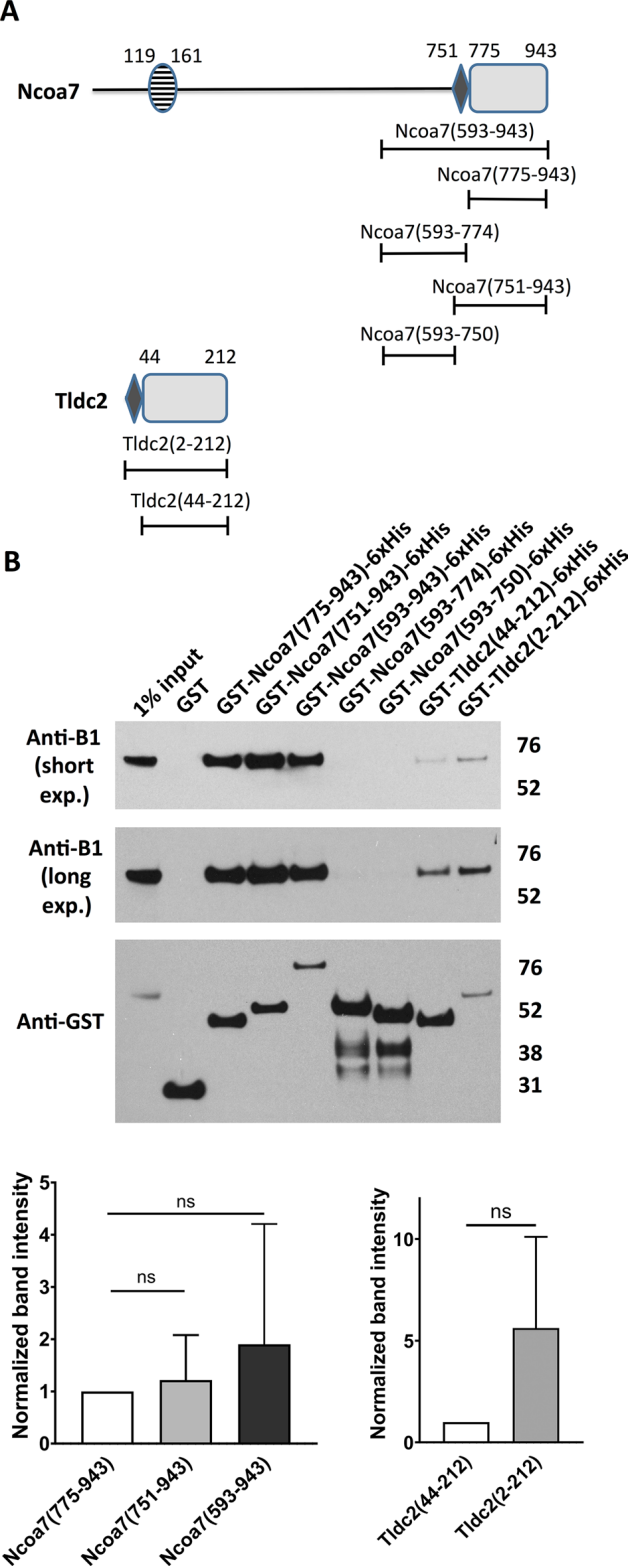
A poly-E rich motif, located upstream of the TLDc domain in Ncoa7 and Tldc2, enhances their interaction with the V-ATPase, but is not sufficient to produce significant interaction with the V-ATPase by itself. (**A**) Schematic representation of the domain architecture of the Ncoa7 and Tldc2 proteins and the constructs used to study the role of the poly-E rich motifs from Ncoa7 and Tldc2 in their interaction with V-ATPase. The conserved regions of the TLDc domains are shown as grey rectangles and poly-E rich motifs as small black rhombuses. Other details and boundaries of the domains and constructs are as indicated in Fig. 1. (**B**) Anti-B1 and anti-GST western blots of a representative GST pull-down assay, using the purified GST-tagged TLDc domains of Ncoa7 (775–943) and Tldc2 (44–212) without poly-E rich motifs, Ncoa7 (751–943), Ncoa7 (593–943) and Tldc2 (2–212) proteins containing both the poly-E rich motif and the TLDc domain, Ncoa7 (593–750) lacking both the poly-E rich motif and the TLDc domain, and finally Ncoa7(593–774), containing the poly-E rich motif but lacking the TLDc domain. A longer exposure of anti-B1 blot is shown to better visualize a relatively weaker ~ 55 kDa band of B1 subunit of V-ATPase in Tldc2 (44–212) and Tldc2 (2–212) GST pull-downs. GST only pull-down was used as a negative control; anti-GST blot was used as a loading control for comparison between samples. This experiment was repeated three times with similar results. (**C**) Quantification of western blotting results by band densitometry analysis. Anti-B1 band densities were divided by anti-GST band densities and then normalized relative to the Ncoa7 (775–943) B1/GST ratio for all Ncoa7 constructs or relative to Tldc2 (44–212) B1/GST ratio for Tldc2 constructs. All values are means ± SE. ns—non significant, P = 0.6852 for Ncoa7 (775–943) vs. Ncoa7 (751–943), P = 0.5116 for Ncoa7 (775–943) vs. Ncoa7 (593–943), P = 0.3611 for Tldc2 (44–212) vs. Tldc2 (2–212), by t-test. Note, that Ncoa7 (751–943), Ncoa7 (593–943) and Tldc2 (2–212) constructs with both poly-E rich motif and TLDc domain show a trend toward more efficient pulling down the B1 subunit of V-ATPase in comparison with the TLDc only domains of Ncoa7 (775–943) and Tldc2 (44–212). However, the Ncoa7 construct (593–774) containing the poly-E rich motif but lacking the TLDc domain did not pull down the B1 subunit, at all.

Both Ncoa7 and Tldc2, but not Oxr1, Tbc1d24 or Tldc1, contain a distinct poly-E rich motif upstream of their TLDc domains (Figs. S1 and 5A). We hypothesized that this poly-E rich motif could potentially contribute to the interaction of Ncoa7 and Tldc2 with the V-ATPase. To explore this possibility, we expressed and purified the following constructs: (1) Ncoa7 (593–750), lacking both the poly-E rich motif and the TLDc domain, (2) Ncoa7 (593–774), containing the poly-E rich motif but lacking the TLDc domain, (3) Ncoa7 (593–943), containing both the poly-E rich motif and the TLDc domain in addition to a portion of the C-terminus, (4) Ncoa7 (751–943), containing only the poly-E rich motif and the TLDc domain, (5) Ncoa7 (775–943), containing only the TLDc domain, (6) Tldc2 (44–212), lacking the poly-E motif, and (7) full-length Tldc2 (2–212), which consists almost entirely of the poly-E rich motif and the TLDc domain (Fig. 5A). These constructs were then used as bait in GST pull-down assays with mouse kidney lysates. Both constructs lacking the TLDc domain, Ncoa7 (593–750) and Ncoa7 (593–774), did not interact with the V-ATPase, indicating that the presence of a poly-E rich motif without a TLDc domain is not sufficient to produce significant interaction with the V-ATPase (Fig. 5B). In contrast, the constructs with both the poly-E motif and the TLDc domain, Ncoa7 (751–943), Ncoa7 (593–943) and Tldc2 (2–212), showed a trend toward stronger interactions with the V-ATPase in comparison with the TLDc domain-only constructs, Ncoa7 (775–943) and Tldc2 (44–212) (Fig. 5B,C). However, the differences were not significant at the P < 0.05 level, due to high variability in the data (Fig. 5C). Therefore, we conclude that the TLDc domain is necessary and sufficient for interaction with the V-ATPase, and that the poly-E domain is not sufficient by itself to interact with the V-ATPase. It may, however, enhance the binding of the TLDc domain, although more work is needed to prove that this is a significant effect.

### Alanine scanning mutagenesis of the evolutionarily conserved amino acids in Ncoa7 revealed that the G815A, G845A and G896A point mutants did not interact with V-ATPase, while the S817A, L926A and E938A mutations decreased the interaction

**Figure 6:**
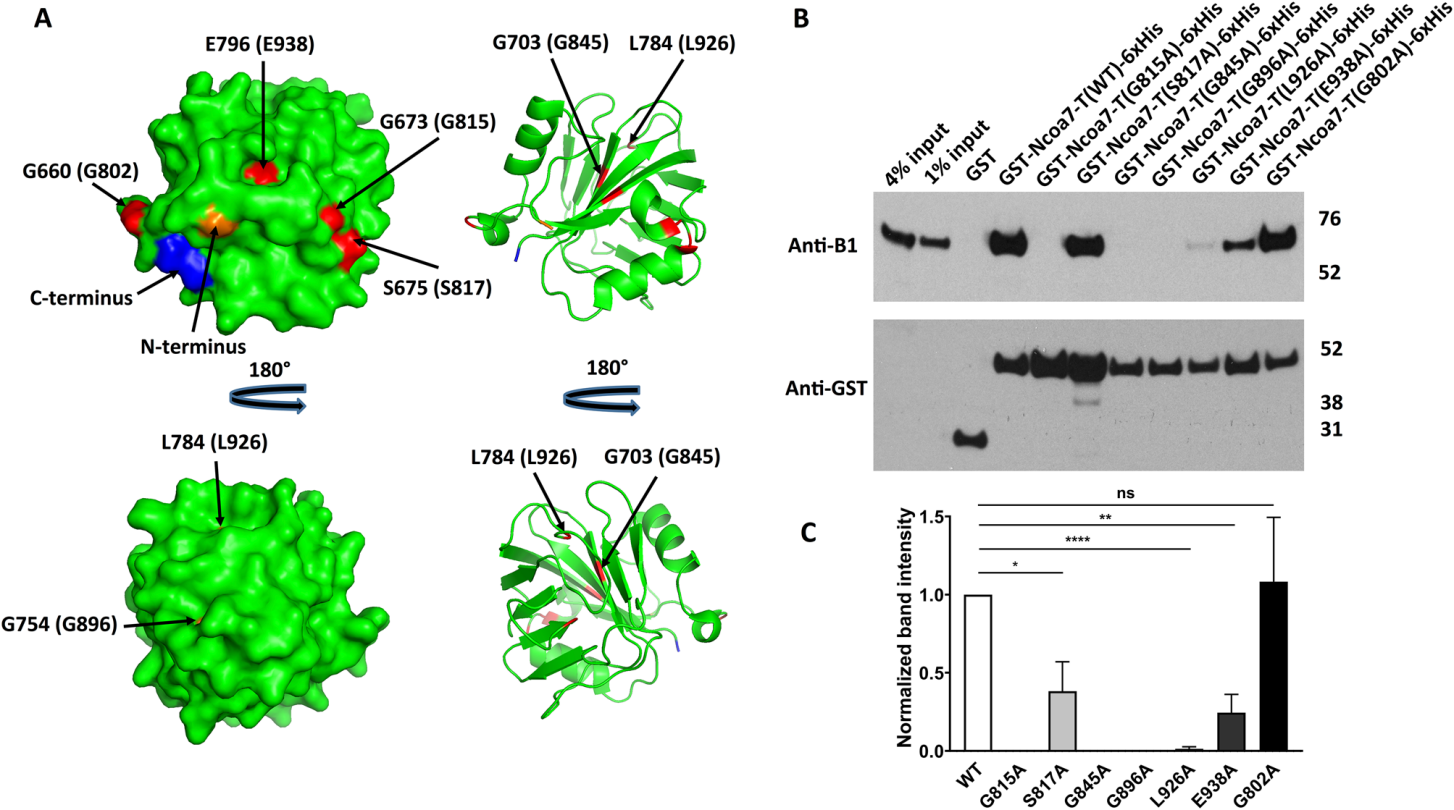
Alanine mutations of the evolutionarily conserved glycines, G815, G845 and G896, completely disrupt Ncoa7 TLDc domain interaction with the V-ATPase, while S817, L926 and E938 mutations show only partial disruption. Mutation of the non-conserved G802 residue (serving as a control) does not inhibit interaction. (**A**) Surface (left) and cartoon (right) representations of the zebrafish OXR2 TLDc domain, created with PyMol (Schrodinger, LLC. 2010. The PyMOL Molecular Graphics System, Version 2.0) using the crystal structure with the protein data bank identifier 4ACJ. The indicated zebrafish OXR2 residues G660, G673 and S675, which correspond to Ncoa7 residues G802, G815 and S817 (shown in parentheses) are exposed on the surface, while residues G703, G754, L784 and E796, which correspond to G845, G896, L926 and E938 in Ncoa7 (shown in parentheses) are buried within the protein. All these residues are colored in red. N-terminal G635 and C-terminal E801 amino acid residues are colored in orange and blue respectively, to better visualize the folding of zebrafish OXR2 TLDc domain. (**B**) Anti-B1 and anti-GST western blots of a representative GST pull-down assay, using the purified GST-tagged G802A, G815A, S817A, G845A, G896A, L926A and E938A mutants of the TLDc domain of Ncoa7 (775–943) as baits and kidney lysate, containing B1 subunit of V-ATPase, as a prey. GST only pull-down was used as a negative control; pull-down with GST-tagged wild-type (WT) TLDc domain of Ncoa7 (775–943) and the non-conserved G802A mutant were used as positive controls. Anti-GST blot was used as a loading control for comparison between samples. This experiment was repeated three times with similar results. (**C**) Quantification of western blotting results by band densitometry analysis. Anti-B1 band densities were divided by anti-GST band densities and then normalized relative to the WT Ncoa7 (775–943) B1/GST ratio. All values are means ± SE. *P = 0.0304; **P = 0.03; ****P = 0.0001, ns—non significant (P = 0.8494), by t-test. Note, that the detrimental effect of G815A, G845A and G896A mutations on interaction does not necessarily correlate with the glycine position in the three-dimensional structure of the protein: while G845 and G896 are buried within the protein, G815 is located on its surface (**A**).

Next, we asked which particular amino acids within the TLDc domain of Ncoa7 are important for its interaction with V-ATPase. Several amino acids in the TLDc domain of Ncoa7 are highly conserved across many species^10^, such as G815, S817, G845, G896, L926 and E938 (numbering is based on protein sequence NP_766083 of the Ncoa7 long isoform). Substitution of each of these amino acids with alanine in the short Ncoa7-B isoform resulted in a less efficient protection against oxidative stress in comparison with wild type isoform, suggesting that these amino acids play an important role in this function of Ncoa7^10^. Therefore, we examined whether these mutations also affect the ability of Ncoa7 to interact with V-ATPase, using the GST pull-down approach described above. In addition, a new alanine mutation of the non-conserved G802, predicted to be present in the unstructured loop on the surface of the Ncoa7 TLDc domain (Fig. 6A), was used as a control mutation that we hypothesized would not affect Ncoa7 interaction with the V-ATPase.

The residues G802, G815, S817, G845, G896, L926 and E938 in mouse Ncoa7 are homologous to the residues G660, G673, S675, G703, G754, L784 and E796 in the zebrafish Oxr2, whose crystal structure was solved^13^ (Fig. 6A). Based on the position of the homologous zebrafish amino acids in the structure, the Ncoa7 residues G802, G815 and S817 are exposed on the surface, while residues G845, G896, L926 and E938 are buried within the protein (Fig. 6A). Mutations of the buried residues are expected to affect the overall TLDc domain structure and stability. In agreement with that, when overexpressed in bacteria the G845A, G896A, L926A and E938A TLDc mutants were mostly insoluble, suggesting that the majority of the mutant protein molecules were not folded properly, and aggregated into the insoluble material (Fig. S3). However, some protein remained soluble and we were able to purify an amount sufficient to perform GST pull-down assays (Fig. S3). In contrast, G802A, G815A and S817A mutations on the protein surface did not affect the overall folding and, when overexpressed in bacteria, were very soluble (Fig. S4). All of these bacterially expressed Ncoa7 TLDc domain mutants were then purified and used as baits in GST pull-down experiments together with the wild type TLDc domain. Interestingly, in the GST pull down experiments, alanine mutations of surface exposed residues G815 and S817, which are located in the same short loop very close to each other on the structure (Fig. 6A), affected interaction with V-ATPase differently: while S817A only partially decreased the interaction, G815A completely abolished it (Fig. 6B,C). As expected, the G802A “control” mutation present in another loop further away from G815 and S817 did not decrease interaction with the V-ATPase at all (Fig. 6B,C). Alanine mutations of the buried G845, G896, L926 and E938 residues, that affected folding of Ncoa7 TLDc domain, all decreased interaction with V-ATPase, but to various degrees: G845A and G896A were most detrimental and no interaction between them and the V-ATPase was detected at all; interaction with L926A was barely detectable, while interaction with E938A was only partially decreased relative to the wild-type TLDc domain (Fig. 6B,C). This may be explained by the fact that in comparison to G845, G896 and L926, E938 is located closer to the C-terminus and to the surface of the protein. Therefore, the E938A mutation may not affect the overall 3D structure and interaction with V-ATPase as much as the three other mutations.

In addition, we performed a computational analysis of the effect of G802A, G815A, S817A, G845A, G896A, L926A and E938A mutations on Ncoa7 TLDc domain stability using PoPMuSiCv3.1 predictive software^27^, as described in methods. The calculated ΔΔG values predict the effect of mutations on thermodynamic stability of TLDc domain: the higher ΔΔG values indicate a greater destabilizing effect of the mutation on the TLDc domain folding. With the exception of L926A mutation, predicted ΔΔG values of less than 1 correlated with a medium to high degree of interaction between the mutants and V-ATPase, while values above 1 correlated with a loss of interaction. However, despite having the largest predicted ΔΔG value, the L926A mutant still interacted with V-ATPase, although very weakly (Fig. 6, Table 1). Overall, we found that the higher ΔΔG values strongly correlate with the loss of interaction of the mutants with the V-ATPase, further supporting the idea that Ncoa7 TLDc domain interaction with V-ATPase relies on the 3D folding stability of the TLDc domain (Table 1).

**Table 1:** Correlation between the predicted effect of the mutations on TLDc domain stability and interaction between the mutants and V-ATPase.

Mutation: mouse Ncoa7/zebrafish Oxr2	ΔΔG, kcal/mol	Predicted relative effect of the mutation on TLDc domain stability	Interaction with V-ATPase (in pull-downs)
G802A/G660A	0.84	Small (< 1)	+++
G815A/G673A	1.97	Medium (> 1, < 2)	−
S817A/S675A	0.71	Small (< 1)	++
G845A/G703A	1.44	Medium (> 1, < 2)	−
G896A/G754A	1.31	Medium (> 1, < 2)	−
L926A/L784A	2.42	Large (> 2)	+
E938A/E796A	0.74	Small (< 1)	++

ΔΔG, the change in the change in Gibbs free energy between the folded and unfolded states (ΔG folding) and the change in ΔG folding when a point mutation is present, were calculated by PoPMuSiCv3.1. The smaller value of ΔΔG predicts smaller effect on TLDc domain stability. −, interaction undetectable; +++, interaction is similar to non-mutated (wild-type) protein; ++ and +, reduced and strongly reduced interaction.

In conclusion, alanine mutations of the evolutionarily conserved amino acids G815, S817, G845, G896, L926 and E938 affected interaction with the V-ATPase differently. The G815A, G845A and G896A mutants of the Ncoa7 TLDc domain did not interact with V-ATPase at all and are, therefore, critical residues for this interaction. S817A, L926A and E938A mutations resulted in a decreased interaction with the V-ATPase, while the non-conserved control G802A mutation interacted with V-ATPase to a similar degree as the wild type construct. Alanine mutations of the buried residues G845, G896, L926 and E938 also affected proper folding of TLDc domain and decreased interaction with V-ATPase, suggesting that the overall native three-dimensional structure of the TLDc domain is necessary for its interaction with V-ATPase.

## Discussion

Our principal finding is that all five members of the TLDc family of proteins interact with the V-ATPase, using a combination of pull-down and co-IP assays. In detailed studies using purified domains of these proteins, and site-specific mutagenesis of the TLDc domain of Ncoa7, we found that this interaction depends on distinct amino acid residues within the TLDc domain itself.

In our previous proteomics study, V-ATPase interacting proteins were co-immunoprecipitated and then identified by mass-spectrometry^9^. In this way, we identified Ncoa7 and Oxr1 as V-ATPase interacting proteins with high interaction scores, but Tbc1d24, although detected, did not reach the high threshold for interaction that we applied in that study, and Tldc1 and Tldc2 were not detected at all^9^. It is known that mass-spectrometry is sometimes unable to identify particular proteins^28^, which is why an alternative method of identification, such as western blotting, is important to use, especially if there is a strong indication for a potential interaction. Indeed, in our current study, we were able to clearly identify Tbc1d24 among the proteins co-immunoprecipitated with V-ATPase from kidney, using anti-Tbc1d24 antibodies for detection by western blotting (Fig. 3B). The low level of expression of Tldc1 and Tldc2 probably explains why we were not able to co-IP them from kidney, but they were clearly detected when overexpressed and co-IP’ed from HEK293 cell lysates.

Furthermore, we also found here that Tbc1d24 and Tldc2, but not Tldc1 interacted preferentially with the V-ATPase B1 but not the B2 subunit in co-immunoprecipitation studies using kidney lysates. V-ATPase is a multifunctional enzyme and plays the very specific role in kidney to maintain acid–base homeostasis by secreting excess protons into the urine. To better perform this function, kidney intercalated cells express several highly specific subunit isoforms of V-ATPase, including the B1 subunit. Likewise, they also express higher levels of Tbc1d24 and Tldc2, which, as we found in this study, preferentially interact with the V-ATPase B1 subunit and, therefore, may play a critical role in function of the kidney-specific V-ATPase. On the other hand, interaction of more ubiquitously expressed Tldc1 with both B1 and B2 subunit isoforms of V-ATPase suggests that it also plays a significant role in the function of ubiquitous V-ATPases, such as acidification of lysosomes and other intracellular vesicles.

While we found by co-IP that all five TLDc family members interact with the V-ATPase, by GST pull-down we were able to narrow down the interaction site to the TLDc domain in only three of them—Ncoa7, Oxr1 and Tldc2, but could not detect interaction between V-ATPase and the purified TLDc domains of Tbc1d24 and Tldc1. According to multiple sequence alignment analysis, Ncoa7 is most closely related to Oxr1, followed by Tldc2 and Tbc1d24, while Tldc1 is the most evolutionarily distant protein from Ncoa7 (Fig. S1B,C). It is, therefore, possible that the TLDc domains of Tbc1d24 and Tldc1 evolved in a way that makes their interaction with the V-ATPase weaker than that of the other TLDc domains. In addition, when expressed in bacteria the TLDc domains of Tbc1d24 and Tldc1 could be folded improperly, or lack critical post-translational modifications, which are well-known limitations of bacterial expression systems^29^.

In comparison with the TLDc domains of other TLDc protein family members, the TLDc domains of Tbc1d24 and Tldc1 contain a non-conserved insertion and a long C-terminal extension respectively, that protrude from their TLDc domains (Fig. S1A) and could potentially inhibit interaction with V-ATPase. However, the truncated versions of the TLDc domains of Tbc1d24 and Tldc1, lacking these protrusions, still did not interact with V-ATPase in our hands (Fig. 2B), suggesting that they are not inhibitory. It is again possible that both full and truncated versions of the Tbc1d24 and Tldc1 TLDc domains are not folded and/or modified correctly, when expressed in bacterial cells.

Originally, the TLDc domain was discovered by computational analysis and was predicted to be catalytic. Although it was shown to be protective against oxidative stress in multiple studies, no direct enzymatic activity was confirmed for this domain^10,30,31^. To the best of our knowledge, our study is the first systematic report of TLDc as a protein–protein interaction domain. In addition to the TLDc domain, we also found that a poly-E rich motif, present only in Ncoa7 and Tldc2, showed a trend, albeit not reaching statistical significance, to enhance their interaction with the V-ATPase. However, this trend occurred only in the presence of the TLDc domain and, thus, can be considered as an accessory interaction site. Similar poly-E repeats are relatively common in proteins, but their function is unknown^32^. Recently, large-scale analysis of bacterial and eukaryotic genomes revealed, that proteins containing homo-repeats, including poly-E, have a large number of interactions^33^, but as far as we know it has never been shown previously that poly-E rich motifs are directly involved in protein–protein interactions. In summary, we found that TLDc is a novel protein–protein interaction domain and is the principal site of interaction with the V-ATPase in Ncoa7, Oxr1, and Tldc2, while poly-E repeats may play a previously unrecognized role as protein–protein interaction enhancers in Ncoa7 and Tldc2, although more work is now needed to confirm this finding.

Finally, in a more detailed study of the TLDc domain of Ncoa7, we found that mutations in the conserved glycines G815A, G845A and G896A, relative to conserved non-glycine mutations S817A, L926A and E938A, caused the most detrimental effect on TLDc domain binding to the V-ATPase. Glycine residues are more conformationally flexible than other amino acids, including alanine. If they are present in regions that require flexibility, such as tight turns, their mutations to other amino acids could lead to critical changes in structure^34^. This is most likely the case for the first G815A mutation that we studied. It was published previously that only the glycine at this position 815 (or position 93, if numbering is based on the short isoform Ncoa7, Ncoa7-B) is flexible enough to accommodate main chain torsion angles required for the bend between two adjacent alpha helixes (Fig. 6A)^10^. Therefore, the G815A mutation will affect local folding that seems to be important for interaction with the V-ATPase. Moreover, this result also suggests, that G815 and/or surrounding amino acids can be directly involved in the interaction with V-ATPase. The second glycine-to-alanine mutation in our study was G485. It is deeply buried within the protein (Fig. 6A), and mutation to the bulkier and less flexible alanine residue would cause steric clashes, affecting overall protein folding and, therefore, interaction with the V-ATPase. Finally, the third analyzed glycine, G896, is also buried within the protein, but is closer to the surface than G845 (Fig. 6A). G896 is located at the beginning of the conserved glycine-rich GGGGGRFG bend (896–903 a.a. in mouse Ncoa7) connecting two beta strands (Figs. 6A and S1). Mutation of this glycine to a conformationally more restrained alanine residue may destabilize this bend or the overall 3-D structure, resulting in an inability to interact with the V-ATPase.

Interestingly the first studied mutation, G815A (in mouse Ncoa7), corresponds to G376A in human Tbc1d24 (Fig. S1), a recurrent pathological mutation in Tbc1d24 that causes neurological diseases including epilepsy^16^. Another recurrent Tbc1d24 pathological mutation A515V corresponds to the position G903 in Ncoa7 at the end of the above-mentioned glycine-rich bend, close to G896 (Fig. S1)^16^. There are also rarer pathological mutations in this region, A500V, G501R, G511R, that are clustered between amino acids 500 and 511 in Tbc1d24 (corresponding to 887–898 aa in mouse Ncoa7) (Fig. S1)^35^. Thus, the G815A and G896A mutations that affect the interaction of the TLDc domain of Ncoa7 with the V-ATPase correspond to pathological mutation G376A and to the G509 position in the region containing a large number of known disease-causing mutations in Tbc1d24.

In conclusion, we describe here a new class of V-ATPase interacting proteins and provide information on the molecular details of their interaction. Our prior data show that one of these TLDc proteins, Ncoa7, is involved in regulating V-ATPase expression and the proton-pumping activity of specialized renal epithelial cells^20^. The association between other family members and the V-ATPase that has been uncovered here could help explain the pathogenesis of some human diseases that involve the TLDc protein family, as well as providing insights into the potential interaction of these proteins with the V-ATPase in conditions of oxidative stress in cells.

## Methods

### Mice

C57BL/6J (wild-type) mice (Jackson Laboratory, Bar Harbor, ME), and B1 promoter EGFP-transgenic mice^36^ were housed under standard conditions and maintained on a standard diet. All animal studies were approved by the Massachusetts General Hospital Subcommittee on Research Animal Care, in accordance with the National Institutes of Health (Bethesda, MD), Department of Agriculture, and Association for the Assessment and Accreditation of Laboratory Animal Care requirements, and followed the ARRIVE guidelines^37^.

### Antibodies

Rabbit and chicken polyclonal antibodies against the B1 (anti-B1) and B2 (anti-B2) subunits of V-ATPase, chicken polyclonal antibodies against the A subunit of V-ATPase (anti-A) were produced, affinity purified and characterized previously in our laboratory^9,38–40^. Commercial, affinity purified antibodies used in the study were: mouse monoclonal anti-GST (B-14), (Santa Cruz Biotechnology, Dallas, TX, sc-138, 1:2500 for western blotting (WB)), rabbit polyclonal anti-Tbc1d24 (Abcam, Cambridge, MA, ab101933, lot GR228820-6, 1 µg/ml for WB, 5 µg/ml for immunofluorescence (IF)), rabbit polyclonal anti-Tldc1 (ProSci, Fort Collins, CO, 55–233, 1 µg/ml for WB), rabbit polyclonal anti-Tldc2 (Origene, Rockville, MD, TA333563, 1 µg/ml for WB), HRP-conjugated rabbit monoclonal anti-β-Actin (13E5) (Cell Signaling Technology, Danvers, MA, 5125, 1:1000 for WB), rabbit monoclonal anti-B2 (D3O7Q) (Cell Signaling Technology, 14488), rabbit monoclonal isotype control (DA1E) (Cell Signaling Technology, 3900) and HRP-conjugated anti-HA (Roche, San Francisco, CA, 12013819001, 1:5000 for WB). The specificity of anti-Tbc1d24, anti-Tldc1 and anti-Tldc2 antibodies for the corresponding mouse proteins was validated in this study by western blot analysis using protein lysates from overexpressing cells. The following secondary antibodies were used for WB: HRP-conjugated mouse anti-rabbit IgG (Light-Chain Specific) (D4W3E) (Cell Signaling Technology, 93702, 1:1000), preadsorbed HRP-conjugated goat anti-rabbit IgG (Abcam, ab97080, 1:5000), HRP-conjugated sheep anti-mouse IgG (Cytiva, Marlborough, MA, 45-000-692, 1:5000). The following secondary antibodies were used for IF: highly cross-adsorbed Alexa Fluor^®^ Plus 555-conjugated donkey anti-rabbit IgG antibody (Invitrogen/Thermo Fisher Scientific, Waltham, MA, 1:600) and the cross-adsorbed Alexa Fluor^®^ Plus 488-conjugated goat anti-chicken IgY antibody (Invitrogen/Thermo Fisher Scientific, 1:600).

### DNA constructs, mutagenesis, recombinant protein expression, and purification

The constructs for mammalian expression of HA-tagged full-length mouse Oxr1 (NCBI accession number NM_130885), Ncoa7 (NM_172495), Tbc1d24 (NM_001163847), Tldc1 (NM_028883), and Tldc2 (NM_001177439), and HA-tagged G815A, S817A, G845A, G896A, L926A and E938A mutants of Ncoa7-B short isoform (note, that here the numbering is based on the protein sequence NP_766083 of the Ncoa7 long isoform), all cloned into a pcDNA3 vector, have been described previously^10^ and were generously provided by Peter L. Oliver (University of Oxford, Oxford, United Kingdom).

These constructs were used as templates to amplify DNA encoding the N-terminal Ncoa7(2–353), middle Ncoa7(354–592), C-terminal Ncoa7(593–943); TLDc domains Ncoa7(775–943), Oxr1(698–866), Tbc1d24(336–561), Tldc1(235–455) and Tldc2(44–212), truncated versions of Tbc1d24 and Tldc1 TLDc domains Tbc1d24(336-446_496-556) and mTldc1(235–410); Ncoa7(593–750), lacking both the poly-E rich motif and the TLDc domain, Ncoa7(593–774), containing the poly-E rich motif but lacking the TLDc domain, Ncoa7(751–943), containing both the poly-E rich motif and the TLDc domain; full-length Tldc2(2–212) and full-length Tldc1 (2–455), and finally mutants G815A, S817A, G845A, G896A, L926A and E938A of Ncoa7 TLDc domain (Ncoa7-T or Ncoa7(775–943)). A new alanine mutation of the non-conserved G802 was introduced into the wild-type Ncoa7(775–943) construct using QuikChange Site-Directed Mutagenesis Kit from Agilent Technologies (Santa Clara, CA). Note, that all numbering is based on the longest known isoform of each protein: NCBI accession numbers NP_766083 (Ncoa7), NP_001345906 (Oxr1), NP_001157319 (Tbc1d24), NP_083159 (Tldc1) and NP_001170910 (Tldc2). All DNAs were amplified using PfuUltra II Fusion HotStart DNA Polymerase (Agilent Technologies), subcloned into a pGEX-6P-1 vector (Cytiva) in frame with an N-terminal GST-tag and adding a C-terminal 6XHis tag. All constructs were verified by sequencing. All recombinant GST- and 6XHis-tagged proteins were successfully expressed in *Escherichia coli* BL21(DE3) cells and affinity purified using TALON beads (Clontech, Mountain View, CA) according to the manufacturer’s instructions.

### Preparation of mouse kidney and HEK 293T and M-1 cell lysates

Wild type C57BL/6J adult mice were anesthetized with sodium pentobarbital (Nembutal, Abbott Laboratories, Abbott Park, IL, 50 mg/kg body weight, intraperitoneally) and phosphate-buffered saline (PBS) was perfused at a constant rate of 17 ml/min through the cardiac left ventricle to clear the organs of blood. Kidneys were dissected and immediately homogenized in ice-cold lysis buffer (25 mM Tris–HCl, pH 7.4; 150 mM NaCl; 5 mM EDTA, 1% Triton X-100), containing Complete Protease inhibitor cocktail (Roche Applied Science, Indianapolis, IN). Lysates were then clarified by centrifugation followed by filtration through a 0.2 μm Acrodisc Syringe Filter (PALL Life Sciences, Port Washington, NY, PN4454) and either used immediately or aliquoted and kept frozen at − 80 °C until the time of experiment. For some experiments kidney medullas were dissected first and kidney medullary lysates were prepared the same way. HEK293T and M-1 cells were lysed and clarified also the same way. Immediately prior to GST pull-down and co-immunoprecipitation experiments kidney and HEK293T lysates were pre-absorbed using glutathione-Sepharose 4B beads or Protein A Agarose Beads (Cell Signaling Technology) respectively.

### GST pull-down assay

Approximately 14 pmol of each purified GST-tagged protein were immobilized on glutathione-Sepharose 4B beads (Cytiva) and then incubated with the mouse kidney lysate, prepared as described above. Unbound proteins were removed by washing the beads; bound proteins were eluted in NuPAGE (Invitrogen/Thermo Fisher Scientific) sample buffer, separated by NuPAGE and analyzed by western blotting using rabbit polyclonal anti-B1 antibodies, followed by anti-GST antibodies as a loading control. All pull-down experiments were repeated at least three times with similar results, and representative data are shown.

### Co-immunoprecipitation

6 µg of rabbit monoclonal anti-B2 Abs, 3.5 µg of rabbit polyclonal anti-B1 antibodies or 6 µg of rabbit monoclonal isotype control antibodies were incubated with 0.5 mg of mouse kidney or mixed kidney/HEK293T lysates, prepared as described above. Preformed antibody-protein complexes were then bound to protein A agarose beads (Cell Signaling Technology). Unbound material was removed by washing the beads and bound complexes were eluted in NuPAGE (Invitrogen/Thermo Fisher Scientific) sample buffer, separated by NuPAGE and analyzed by western blotting using anti-Tbc1d24, anti-Tldc1, anti-Tldc2, or anti-HA antibodies, followed by chicken anti-B1 and anti-B2 antibodies to confirm the immunoprecipitation of B1 and B2 subunits of V-ATPase. All co-immunoprecipitation experiments were repeated at least three times with similar results, and representative data are shown.

### Cell culture, plasmid and siRNA transfections

For *Tbc1d24, Tldc1 and Tldc2* overexpression experiments, human embryonic kidney HEK-293T cells (ATCC^®^ CRL-3216™, American Type Culture Collection (ATCC), Manassas, VA) were transiently transfected with pCDNA3-based plasmids, expressing HA-tagged full-length mouse Tbc1d24, Tldc1 and Tldc2, using Lipofectamine 2000 transfection reagent (ThermoFisher Scientific) according to the manufacturer’s instructions. For *Tbc1d24* knockdown experiments, Stealth pre-designed siRNAs (set of three) MSS277591, MSS277592, MSS277593, each targeting three different regions of mouse Tbc1d24 mRNAs and one nontargeting Stealth siRNA (negative control) were purchased from ThermoFisher Scientific. Mouse kidney cortical collecting duct M-1 cells (ATCC^®^ CRL-2038™, ATCC) were transiently co-transfected with a pCDNA3-based plasmid, expressing HA-tagged full-length mouse Tbc1d24, with or without a mixture of all three Tbc1d24 siRNAs (40 nM each) or 120 nM of negative control siRNA using Lipofectamine 2000 transfection reagent (Invitrogen). Seventy-two hours after transfection, cells were lysed and analyzed by western blotting.

### Immunohistochemistry and fluorescence microscopy

Fixation of C57BL/6J (wild-type) and B1 promoter EGFP-transgenic 2-month-old mouse kidneys, preparation and storage of cryostat sections, rehydration, antigen retrieval, and incubation with primary and secondary antibodies were performed as previously described^41^. For double immunostaining, we used concurrently the affinity-purified rabbit polyclonal anti-Tbc1d24 antibody at 1:100 and an affinity-purified chicken polyclonal anti-V-ATPase A subunit antibody at 1:400. The secondary cross-adsorbed Alexa Fluor^®^ Plus 555-conjugated donkey anti-rabbit IgG antibody and the secondary cross-adsorbed Alexa Fluor^®^ Plus 488-conjugated goat anti-chicken IgY antibodies (both from Invitrogen/Thermo Fisher Scientific) were used at 1:600 dilution or 3.3 µg/ml. Immunostained kidneys were imaged with a Zeiss LSM 800 Airyscan confocal microscope (Carl Zeiss Microscopy, Thornwood, NY), controlled by ZEN 2 (blue edition) software (Carl Zeiss Microscopy). All immunostaining experiments were repeated at least three times with similar results, and representative images are shown.

### Analysis of the effect of mutations on TLDc domain folding stability by computational methods

Effect of G802A, G815A, S817A, G845A, G896A, L926A and E938A mutations on TLDc domain folding stability was predicted by PoPMuSiCv3.1 (https://soft.dezyme.com/login)^27^. Zebrafish OXR2 TLDc domain crystal structure 4ACJ^13^ was used to calculate the change in the change in Gibbs free energy between the folded and unfolded states (ΔG folding) and the change in ΔG folding when a point mutation is present (ΔΔG) values for each mutation.

### Statistical analysis

Statistical significance was determined by two-tailed unpaired t-test using Prism 9 software (GraphPad Software, La Jolla, CA). P < 0.05 was considered significant. Graphs were plotted with Prism 9 software. Experimental values are reported as means ± standard error (SE).

## Supplementary Information

Supplementary Figures.
