# Author Correction: The evolutionary conserved TLDc domain defines a new class of (H^+^)V-ATPase interacting proteins

**DOI:** 10.1038/s41598-021-02955-z

**Published:** 2021-11-30

**Authors:** A. F. Eaton, D. Brown, M. Merkulova

**Affiliations:** 1 Program in Membrane Biology and Division of Nephrology, Massachusetts General Hospital and Harvard Medical School, Boston, MA 02114 USA; 2 Program in Membrane Biology and Division of Nephrology, Massachusetts General Hospital, Simches Research Center, 128 Cambridge St., Boston, MA 02114 USA

Correction to: *Scientific Reports* 10.1038/s41598-021-01809-y, published online 22 November 2021

The original version of this Article contained an error in Figure 6A where the label for the arrow in the lower left corner was omitted.

The original Figure 6 and accompanying legend appear below. The original Article has been corrected.

**Figure 6:**
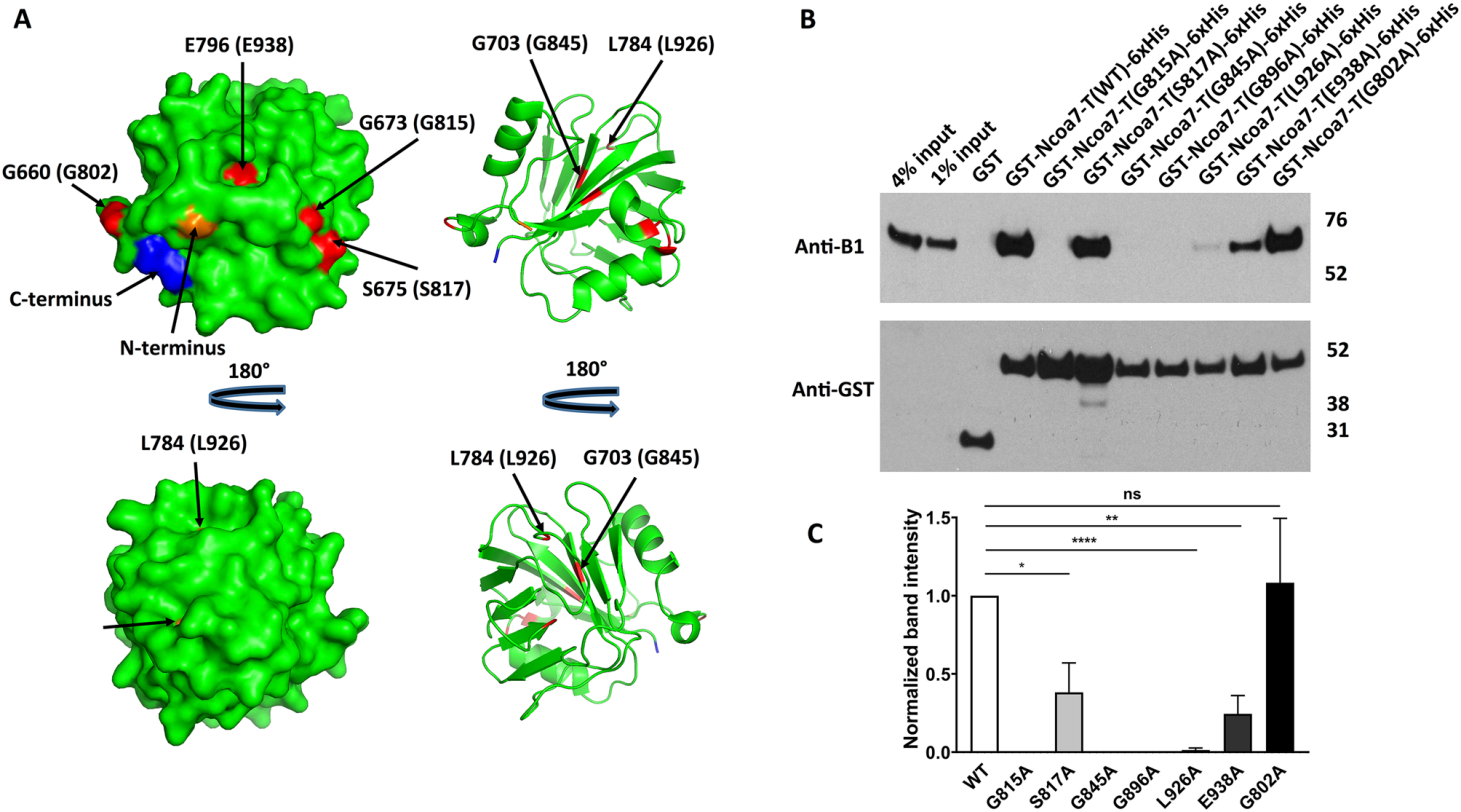
Alanine mutations of the evolutionarily conserved glycines, G815, G845 and G896, completely disrupt Ncoa7 TLDc domain interaction with the V-ATPase, while S817, L926 and E938 mutations show only partial disruption. Mutation of the non-conserved G802 residue (serving as a control) does not inhibit interaction. (**A**) Surface (left) and cartoon (right) representations of the zebrafish OXR2 TLDc domain, created with PyMol (Schrodinger, LLC. 2010. The PyMOL Molecular Graphics System, Version 2.0) using the crystal structure with the protein data bank identifier 4ACJ. The indicated zebrafish OXR2 residues G660, G673 and S675, which correspond to Ncoa7 residues G802, G815 and S817 (shown in parentheses) are exposed on the surface, while residues G703, G754, L784 and E796, which correspond to G845, G896, L926 and E938 in Ncoa7 (shown in parentheses) are buried within the protein. All these residues are colored in red. N-terminal G635 and C-terminal E801 amino acid residues are colored in orange and blue respectively, to better visualize the folding of zebrafish OXR2 TLDc domain. (**B**) Anti-B1 and anti-GST western blots of a representative GST pull-down assay, using the purified GST-tagged G802A, G815A, S817A, G845A, G896A, L926A and E938A mutants of the TLDc domain of Ncoa7 (775–943) as baits and kidney lysate, containing B1 subunit of V-ATPase, as a prey. GST only pull-down was used as a negative control; pull-down with GST-tagged wild-type (WT) TLDc domain of Ncoa7 (775–943) and the non-conserved G802A mutant were used as positive controls. Anti-GST blot was used as a loading control for comparison between samples. This experiment was repeated three times with similar results. (**C**) Quantification of western blotting results by band densitometry analysis. Anti-B1 band densities were divided by anti-GST band densities and then normalized relative to the WT Ncoa7 (775–943) B1/GST ratio. All values are means ± SE. *P = 0.0304; **P = 0.03; ****P = 0.0001, ns—non significant (P = 0.8494), by t-test. Note, that the detrimental effect of G815A, G845A and G896A mutations on interaction does not necessarily correlate with the glycine position in the three-dimensional structure of the protein: while G845 and G896 are buried within the protein, G815 is located on its surface (**A**).

