# Supplementary material for: The evolutionary conserved TLDc domain defines a new class of (H+)V-ATPase interacting proteins
Source: Sci Rep. 2021 Nov 22;11:22654. doi: 10.1038/s41598-021-01809-y (PMC8608904; doi:10.1038/s41598-021-01809-y)
Supplement: Supplementary file 1 — Supplementary Figures. [file 41598_2021_1809_MOESM1_ESM.pdf]

SUPPLEMENTARY INFORMATION

SUPPLEMENTARY FIGURE LEGENDS

Supplementary Figure S1. (A) Multiple sequence alignment of zebrafish OXR2 (OXR2_DANRE), mouse Tldc1 (TLDC1_MOUSE), mouse Tbc1d24 (TBC24_MOUSE), human Tbc1d24 (TBC24_HUMAN), mouse Ncoa7 (NCOA7_MOUSE), mouse Tldc2 (TLDC2_MOUSE) and mouse Oxr1 (OXR1_MOUSE). Numbering is based on the amino acid sequences with NCBI accession numbers AFK08734 (zebrafish OXR2), NP_083159 (mouse Tldc1), NP_001157319 (mouse Tbc1d24), NP_001186036 (human TBC1D24), NP_766083 (mouse Ncoa7), NP_001170910 (mouse Tldc2) and NP_001345906 (mouse Oxr1). Positions of the fully conserved amino acid residues are indicated by an asterisk (*), strongly conserved – by a colon (:) and weakly conserved by a period (.), based on Gonnet PAM 250 matrix scores.

Poly-E rich motifs, located upstream of TLDc domain in Ncoa7 and Tldc2 are colored in cyan. The start of TLDc domain constructs used in this study is indicated by an arrowhead, and the corresponding amino acids residues are colored in red. The Ncoa7 non-conserved amino acid residue G802 and its highly conserved G815, S817, G845, G896, L926 and E938 residues, mutated to alanines in this study, are colored in purple. The conserved glycine-rich GGGGGRFG bend (896-903 aa in mouse Ncoa7) is underlined. The positions of human Tbc1d24 amino acid residues, which recurrent (G376A, A515V) or more rare (A500V, G501R, G511R) pathological mutations cause neurological diseases including epilepsy, are colored in green. Note, that G815 and G896 of mouse Ncoa7 correspond to G376 and G509 of human Tbc1d24 located in the regions with a large number of known pathological Tbc1d24 mutations. Secondary structure assignments are shown above zebrafish OXR2 amino acid sequence, based on the atomic-resolution coordinates of zebrafish OXR2 TLDc domain (protein data bank identifier 4ACJ,[1]) and using DSSP algorithm[2]. DSSP Legend: Empty: no

secondary structure assigned, ***B***: beta bridge, ***S***: bend, ***T***: turn, ***E***: beta strand, ***G***: 3/10-helix, ***H***: alpha helix.

(B) Phylogenetic tree of the mouse Tldc protein family. Note that Tldc1 is the most distantly related protein to Ncoa7, while Oxr1 and Tldc2 are most closely related to Ncoa7.

(C) Percent identity matrix of mouse TLDc proteins, measured as the percent of the number of identical amino acid residues in relation to the length of the alignment for each pair of aligned sequences. Note, that Tldc1 contains the lowest percentage of amino acid residues identical to Ncoa7, or to any other TLDc protein. On the other hand, Tldc2, followed by Oxr1, contains the highest percentage of amino acid residues identical to Ncoa7. Multiple sequence alignment, phylogenetic tree and percent identity matrix were created with Clustal Omega program[3].

Supplementary Figure S2. Endogenous Tldc1 and Tldc2 are not detected in mouse kidney lysates, or in the co-immunoprecipitation with the B1 or B2 subunit of V-ATPase by western blotting.

Proteins were co-immunoprecipitated by anti-B1 and anti-B2 antibodies from mouse total kidney lysates and then analyzed by western blot, using anti-Tldc1 or anti-Tldc2 antibodies. The bands of expected 51 kDa (for Tldc1) and 24 kDa (for Tldc2) molecular massed are not detected in any of the IP lanes. Note that ~50 kDa heavy chains of antibodies, used for co-immunoprecipitation, are visible in the IP lanes, as expected. Anti-B1 and anti-B2 western blots are shown to confirm the successful immunoprecipitation of B1 and B2 subunits of V-ATPase from kidney.

Supplementary Figure S3. High expression level and solubility of bacterially expressed GST- and 6XHis-tagged wild-type (WT) mouse Ncoa7 TLDc domain (Ncoa7-T) and different levels of solubility of its G802A, G815A, S817A, G845A, G896A, L926A and E938A mutants. Total (T) and soluble (S) protein fractions were prepared from *Escherichia coli* BL21(DE3) cells, overexpressing the corresponding fusion protein, resolved by NuPAGE and visualized by Coomassie blue R250 staining. The GST- and 6XHis-tagged Ncoa7-T(WT), Ncoa7-T(G802A),

Ncoa7-T(G815A) and Ncoa7-T(S817A) mutants were highly soluble, while Ncoa7-T(G845A), Ncoa7-T(G896A), Ncoa7-T(L926A) and Ncoa7-T(E938A) mutants were much less soluble, suggesting that they were not folded correctly, when overexpressed in BL21(DE3) cells. Purified bovine serum albumin (BSA) was used as a loading control. The last lane in each gel is a molecular weight standard. The expected molecular weight for all fusion proteins is 47.6 kDa (arrow).

Supplementary References

1 Blaise, M. *et al.* Crystal structure of the TLDc domain of oxidation resistance protein 2 from zebrafish. *Proteins* **80**, 1694-1698, doi:10.1002/prot.24050 (2012).

2 Kabsch, W. & Sander, C. Dictionary of protein secondary structure: pattern recognition of hydrogen-bonded and geometrical features. *Biopolymers* **22**, 2577-2637, doi:10.1002/bip.360221211 (1983).

3 Sievers, F. *et al.* Fast, scalable generation of high-quality protein multiple sequence alignments using Clustal Omega. *Mol Syst Biol* **7**, 539, doi:10.1038/msb.2011.75 (2011).

Supplementary Figure S1

A

```
                                SS  HHHHHHHHTTS GGGTT   EEEEEHHHH S HHHHHHH
OXR2_DANRE     569    QQPEESSEKEWEVVSWTEYHRRIDALNSEDLRSLCKRLQIATKEDVNSKHGTS--ITASL  626
TLDC1_MOUSE    187    QVCDQAMIEEWVFHVPH---VG-MFLSVVVHRGLCLLG------------SSF--DPSTL  228
TBC24_MOUSE    280    VSPEKLLEKAFAIRLFS---RK-------E----IQLLQMANEKALRQKGITVKQKSVSL  325
TBC24_HUMAN    280    VSPEKLLEKAFAIRLFS---RK-------E----IQLLQMANEKALKQKGITVKQKSVSL  325
NCOA7_MOUSE    737    -FFSEPTTKSWEIITVEEAKRRKST---------CSYY----EEEEEEE-----------  771
TLDC2_MOUSE      1    -------MKGWQ--------WRYTQLPTEMEDTLS------GEEGEEEEEEEP--APAPA  37
OXR1_MOUSE     635    PA-GEAAAREWEVVSVAEYHRRIDALNTEELRTLCRRLQITTREDINSKQVAP--AKADL  691
                              . :
```

```
                                ▼      SS  HHHHHHHHTTS GGGTT   EEEEEHHHH S HHHHHHH
OXR2_DANRE     627    EPETFK----PNLNEPSDLLEAEQIEKLAKHLPPRTIGYPWNLAFSTSKHGMSIKTLYRA  682
TLDC1_MOUSE    229    VPECLA----DQGGRFESILDVLSVIYLSSHLAPE-HRQRWRLLFSTQLHGQSFSQLCSH  283
TBC24_MOUSE    326    SKRQFVHLAVHAENFHSEIVSVKEMRDIWSWIPERFALCQPLLLFSSLQHGYSLSRFYFQ  385
TBC24_HUMAN    326    SKRQFVHLAVHAENFRSEIVSVREMRDIWSWVPERFALCQPLLLFSSLQHGYSLARFYFQ  385
NCOA7_MOUSE    772    ---EGL----PILQPHSALLENMHIEQLARRLPARVQGYPWRLAYSTLEHGTSLKTLYRK  824
TLDC2_MOUSE     38    PQDPVE----PQLTEASQVLGASEIKQLSLHLPPRVTGHPWSLVFCTSRDGFSLRRLYRQ  93
OXR1_MOUSE     692    EPESFR----PNLSDPSELLLPDQIEKLTKHLPPRTIGYPWTLVYGTGKHGTSLKTLYRT  747
                                      . ::    :  :   :  .       * : :  .* *:  :
```

```
                      HTT  S EEEEEEEETTS EEEEEEESS          SS B  TT EEEE SSS EEEE
OXR2_DANRE     683    MQDQDSPMLLVIKDSDGQIFGALASEP------FKVSEGFYGTGETFLFTFYPEFEAYKW  736
TLDC1_MOUSE    284    ITSQ-GPSLLVLEDRDGYVFGGFASCSWEVK------PQFQGDNRCFLFSIAPRMATHLH  336
TBC24_MOUSE    386    CEGHE-PTLLLIKTTQKEVCGAYLSTDWSERTKFGGKLGFFGTGECFVFRLQPEVQRYEW  444
TBC24_HUMAN    386    CEGHE-PTLLLIKTTQKEVCGAYLSTDWSERNKFGGKLGFFGTGECFVFRLQPEVQRYEW  444
NCOA7_MOUSE    825    SASLDSPVLLVIKDMDNQIFGAYATHP------FKFSDHYYGTGETFLYTFSPNFKVFKW  878
TLDC2_MOUSE     94    MEGHSGPVLLLLRDQDGQMFGAFSSSA------IRLSKGFYGTGETFLFSFSPQLKVFKW  147
OXR1_MOUSE     748    MTGLDTPVLMVIKDSDGQVFGALASEP------FKVSDGFYGTGETFVFTFCPEFEVFKW  801
                        .   * *:::.  :  : *.  :              : * .. *:: : *..  .
```

```
                       S                                                 S    EEEE
OXR2_DANRE     737    TG-------------------------------------------------DNLFFIKGD  747
TLDC1_MOUSE    337    TGYN-----------------------------------------------NHFMYLNYG  349
TBC24_MOUSE    445    VVIKHPELTKATSLKSSEAAGSSSLISHCSSDPADRLSPFLAARHFNLPSKTESMFMAGG  504
TBC24_HUMAN    445    VVIKHPELTKPPPLMAAEPT--APLSHSASSDPADRLSPFLAARHFNLPSKTESMFMAGG  502
NCOA7_MOUSE    879    SG-------------------------------------------------ENSYFINGD  889
TLDC2_MOUSE    148    TG-------------------------------------------------HNSFFVKGD  158
OXR1_MOUSE     802    TG-------------------------------------------------DNMFFIKGD  812
                                                                          .  ::  .
```

```
                          TTEEEES SSSS SEEEETTS EEEE     BTTTTB  SSSSSEEEEEEEEEEE
OXR2_DANRE     748    ----MDSLAFGGGSGEFGLWLDGDLYHGRN---HSCKTFGNPMLSMKEDFFVQDIEIWSF  800
TLDC1_MOUSE    350    QQTMPNGLGMGGQHHYFGLWVAADFGKGHSKAKPACTTYNSPQLSAQEDFLFDKMEVWGL  409
TBC24_MOUSE    505    ----NDCLIIGGGGGQ-ALYVDGDLNRGRT---GHCDTFNNQPLC-SENFLIAAVEAWGF  555
TBC24_HUMAN    503    ----SDCLIVGGGGGQ-ALYIDGDLNRGRT---SHCDTFNNQPLC-SENFLIAAVEAWGF  553
NCOA7_MOUSE    890    ----ISSLELGGGGGRFGLWLDADLYHGRS---NSCSTFNNDILSKKEDFIVQDLEVWTF  942
TLDC2_MOUSE    159    ----LDSLMMGSGSGQFGLWLDGDLYHGGS---YPCATFNNEVLARREQFCIKELEAWVL  211
OXR1_MOUSE     813    ----MDSLAFGGGGGEFALWLDGDLYHGRS---HSCKTFGNHTLSKKEDFFIQDIEIWAF  865
                           . * .*.     .*:: .*: :* .     * *:..  *.  *:* .  :* * :
```

```
OXR2_DANRE     801    E---------------------------------------------  801
TLDC1_MOUSE    410    GNLLEEYEGKNKKSVLDSNPEARSLLEISGRARHSEGLREVPRDED  455
TBC24_MOUSE    556    QDPDTE----------------------------------------  561
TBC24_HUMAN    554    QDPDTQ----------------------------------------  559
NCOA7_MOUSE    943    E---------------------------------------------  943
TLDC2_MOUSE    212    S---------------------------------------------  212
OXR1_MOUSE     866    E---------------------------------------------  866
```

B

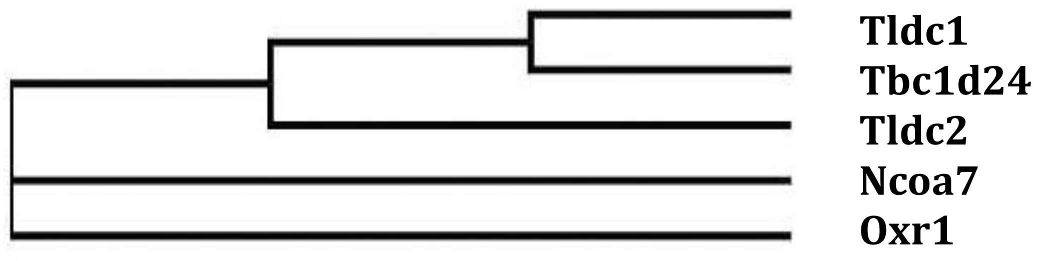

C

	Tldc1	Tbc1d24	Ncoa7	Oxr1	Tldc2
Tldc1	100.00	22.65	22.75	21.04	29.95
Tbc1d24	22.65	100.00	24.70	25.07	30.77
Ncoa7	22.75	24.70	100.00	43.19	48.33
Oxr1	21.04	25.07	43.19	100.00	46.97
Tldc2	29.95	30.77	48.33	46.97	100.00

Supplementary Figure S2

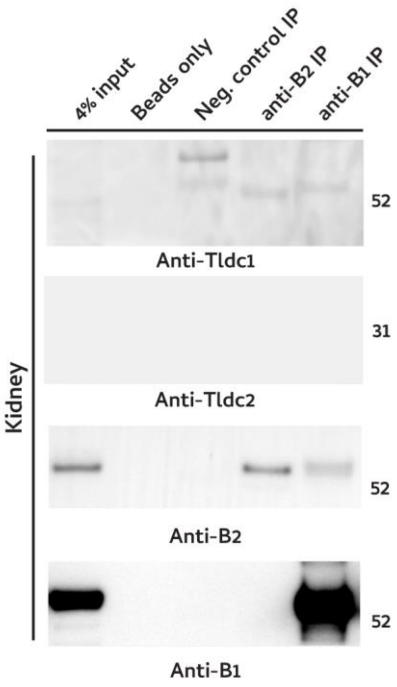

Supplementary Figure S3

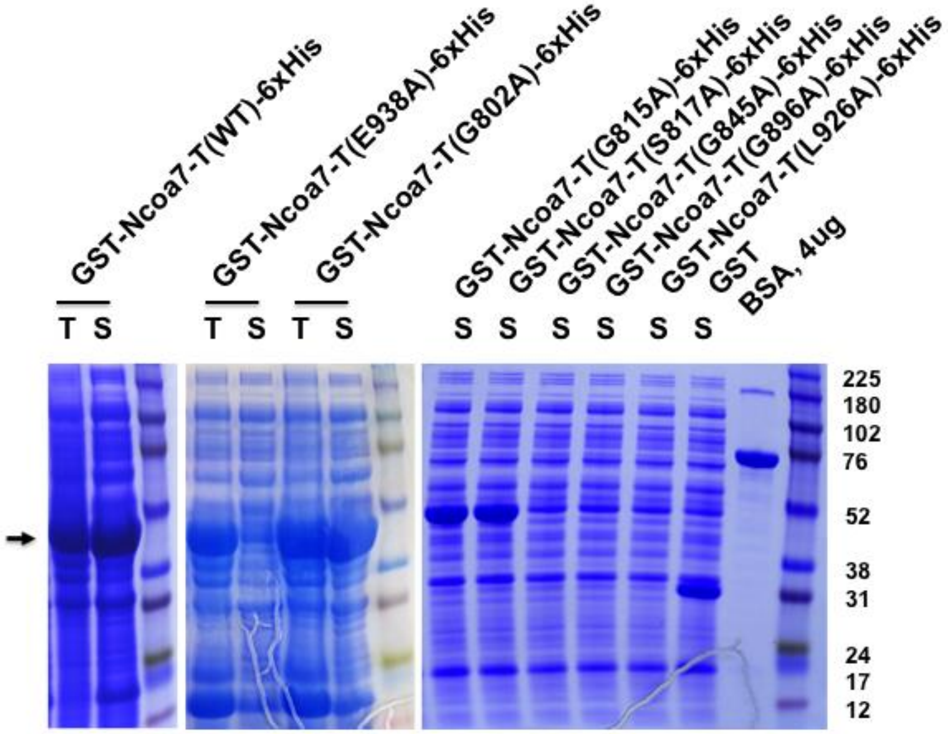

Full-size blots and gels

Fig. 1B. Anti-B1 western blot

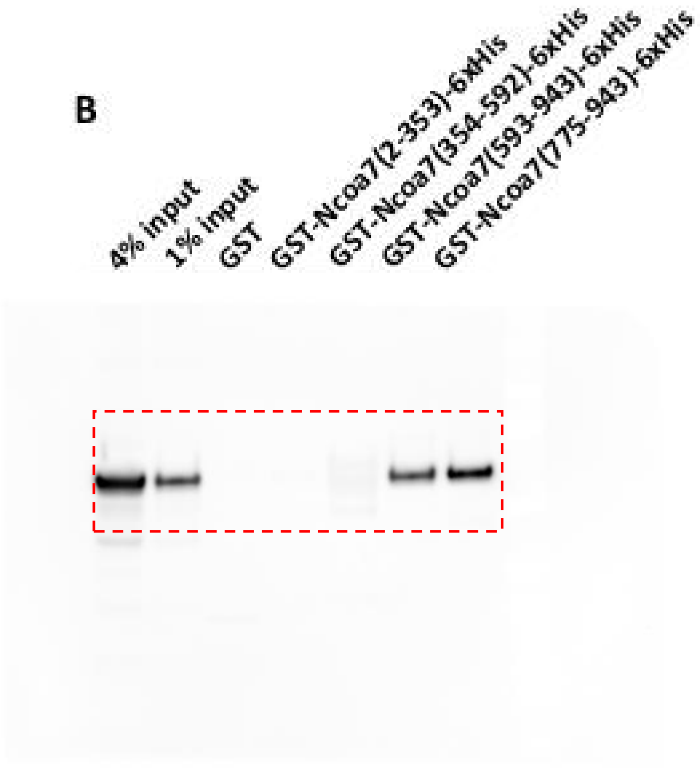

ECL (G:BOX mini imaging system, Syngene, Synoptics)

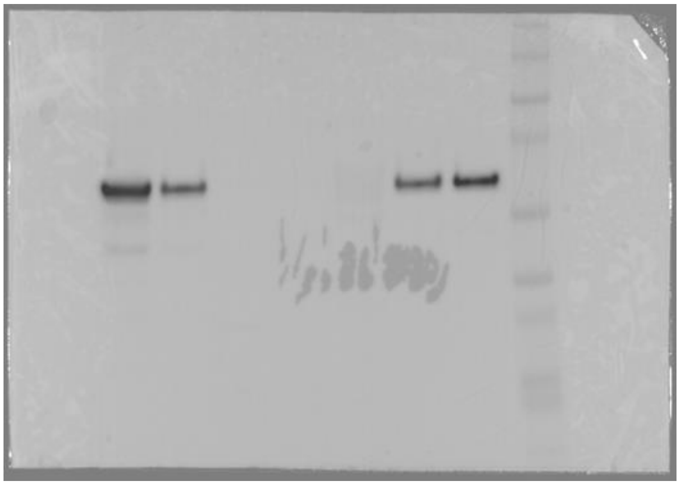

ECL + Visible (G:BOX mini imaging system, Syngene, Synoptics)

Fig. 1B. Anti-GST western blot

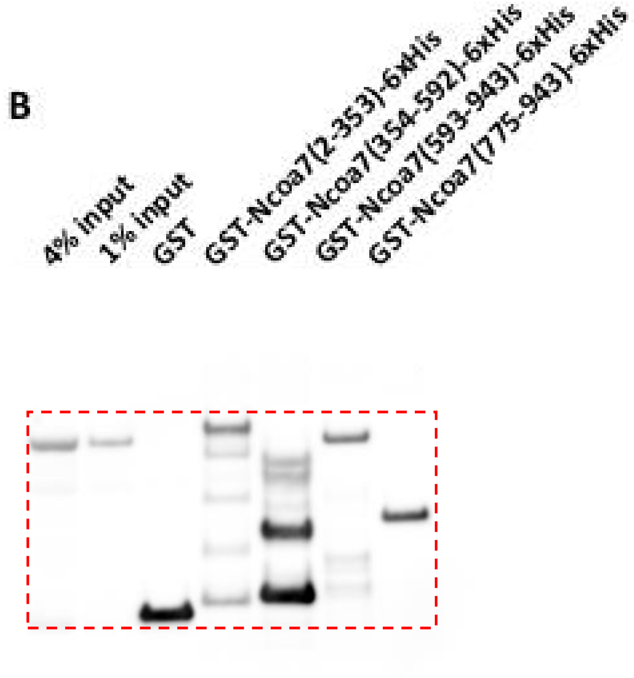

ECL (G:BOX mini imaging system, Syngene, Synoptics)

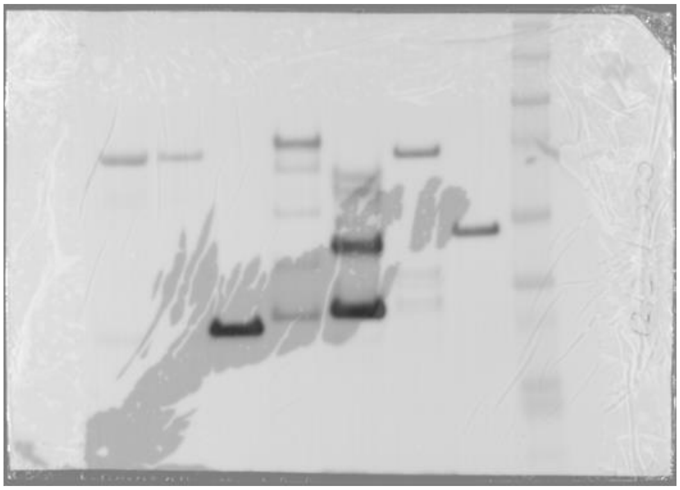

ECL + Visible (G:BOX mini imaging system, Syngene, Synoptics)

Fig. 1C. Anti-B1 western blot

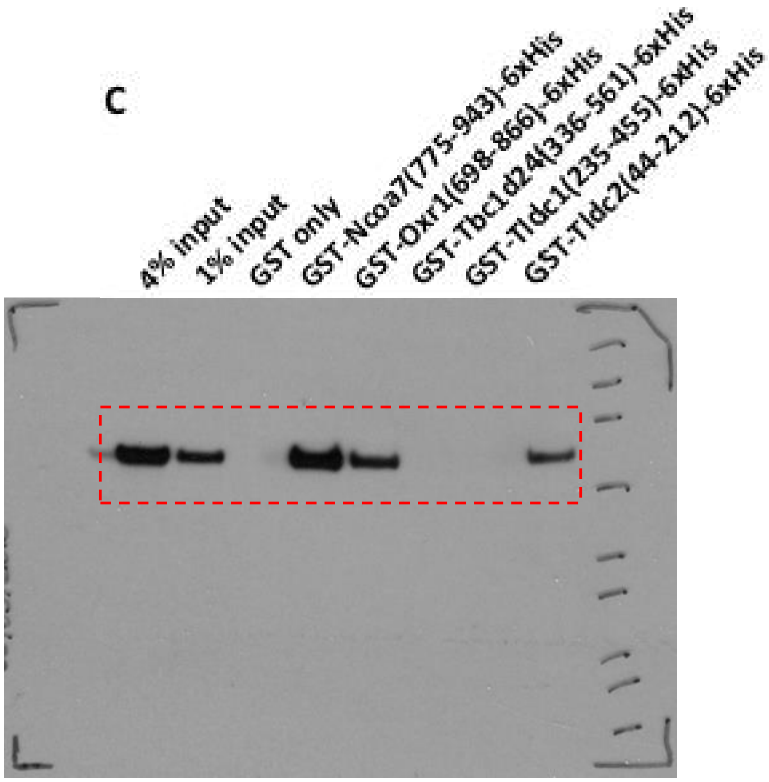

Film

Fig. 1C. Anti-GST western blot

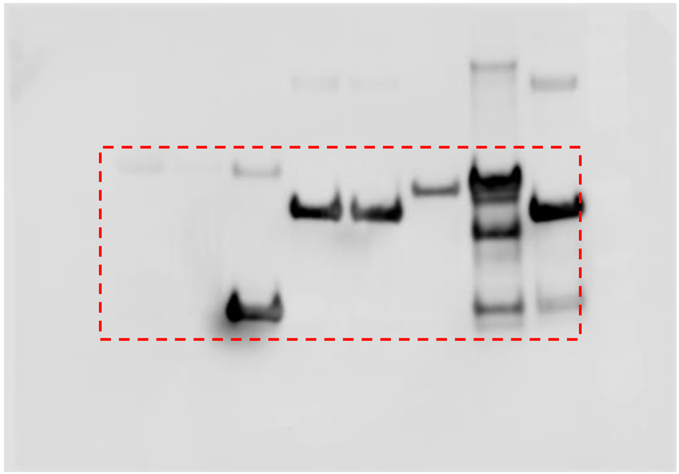

ECL (G:BOX mini imaging system, Syngene, Synoptics)

Fig. 1C. Anti-GST western blot, continued

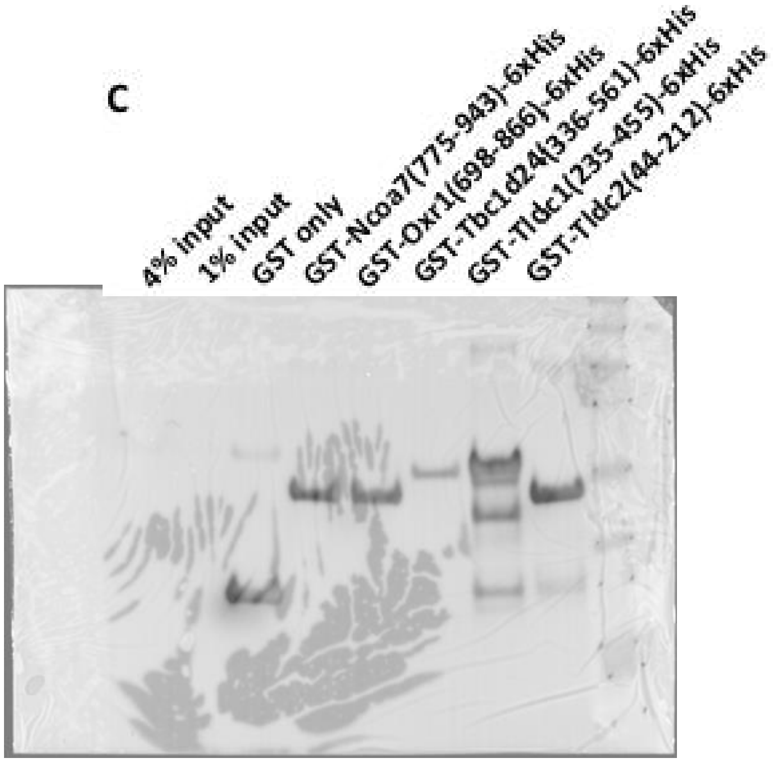

ECL + Visible (G:BOX mini imaging system, Syngene, Synoptics)

Fig. 2B. Anti-B1 western blot

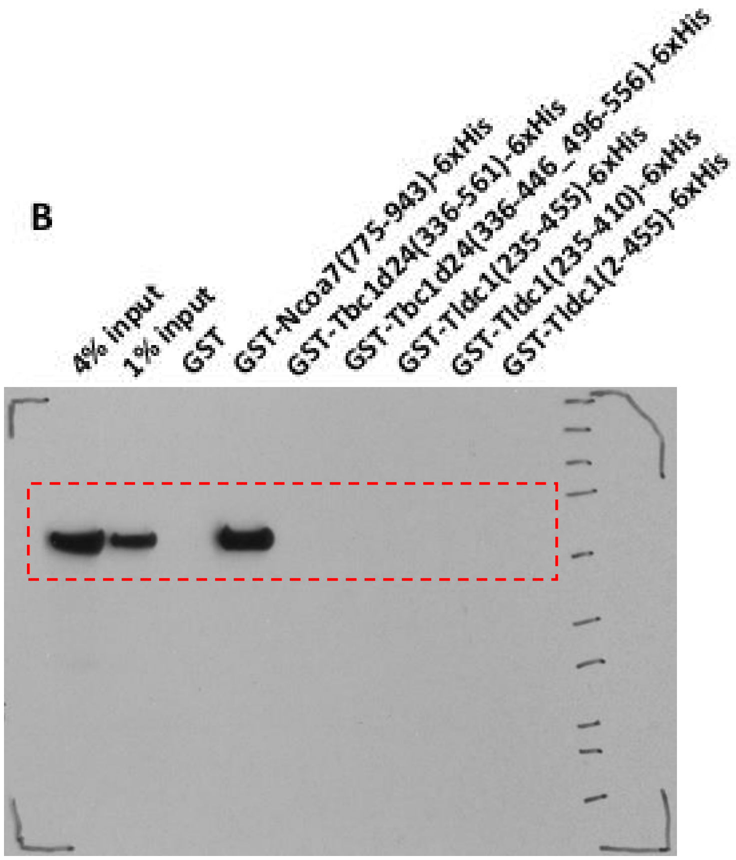

Film

Fig. 2B. Anti-GST western blot

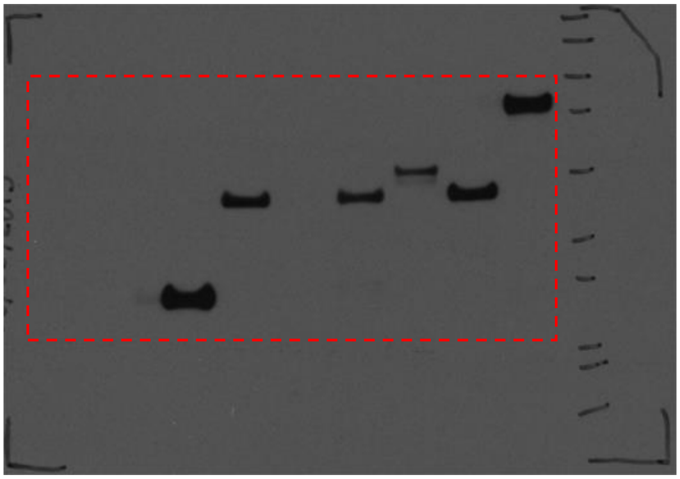

Film, short exposure

Fig. 2B. Anti-GST western blot, continued

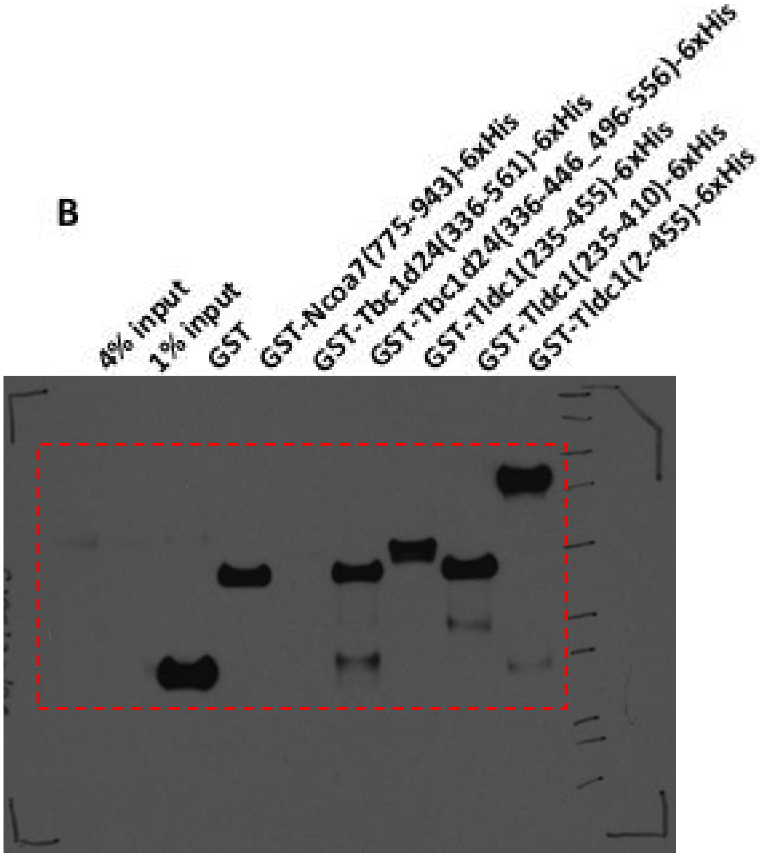

Film, long exposure

Fig. 3A. Anti-Tbc1d24 western blot

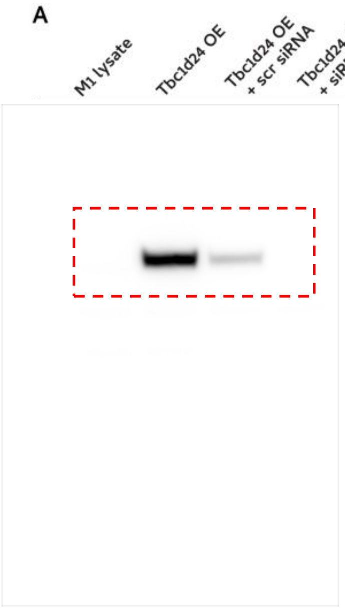

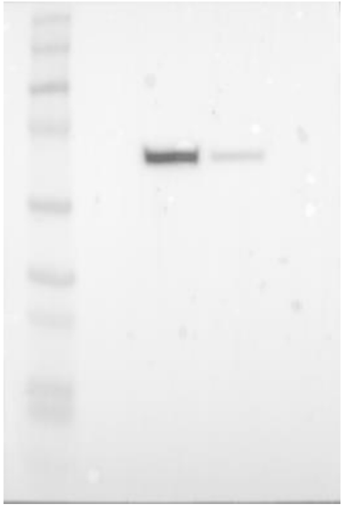

ECL ECL + Visible
(G:BOX mini imaging system, Syngene, Synoptics)

Fig. 3A. Anti-β-actin western blot

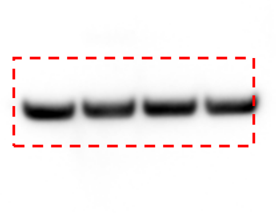

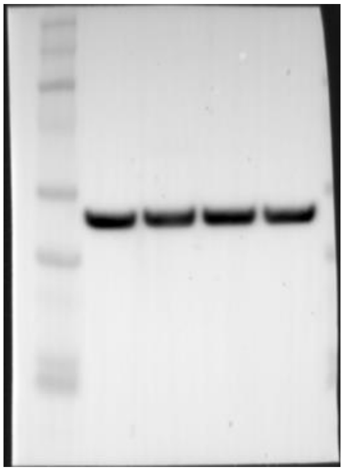

ECL ECL + Visible
(G:BOX mini imaging system, Syngene, Synoptics)

Fig. 3B. Anti-Tbc1d24 western blot

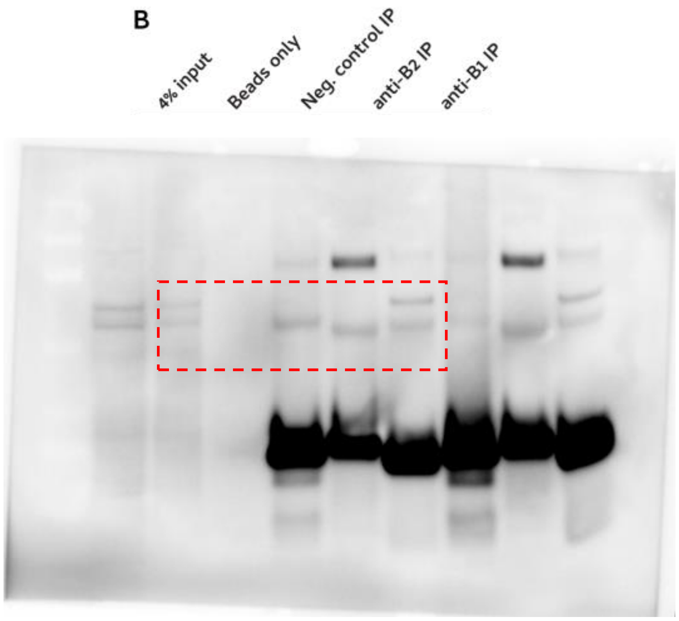

ECL (G:BOX mini imaging system, Syngene, Synoptics)

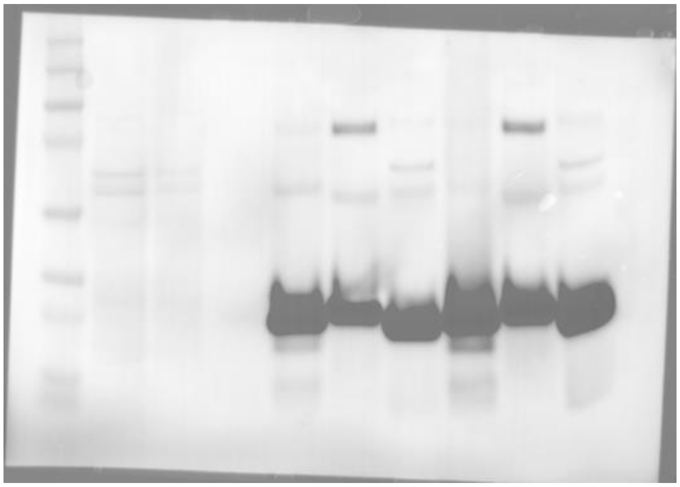

ECL + Visible (G:BOX mini imaging system, Syngene, Synoptics)

Fig. 3B. Anti-B2 western blot

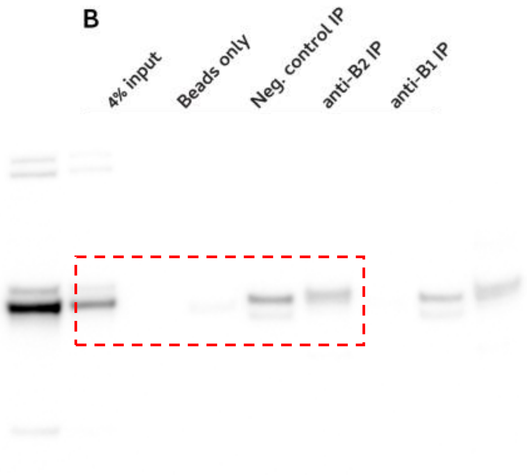

ECL (G:BOX mini imaging system, Syngene, Synoptics)

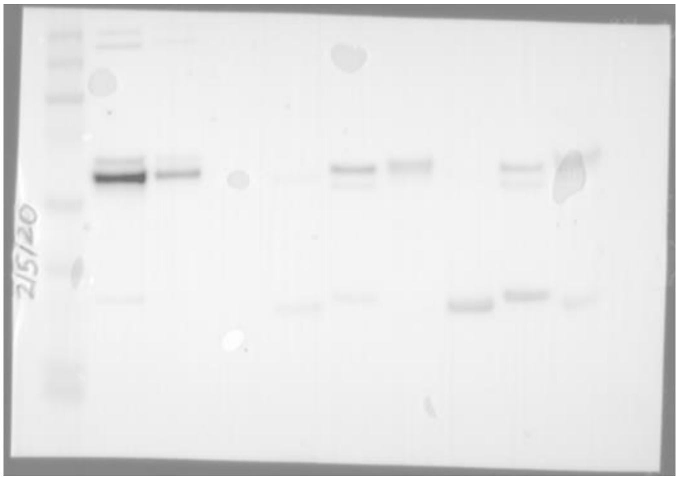

ECL + Visible (G:BOX mini imaging system, Syngene, Synoptics)

Fig. 3B. Anti-B1 western blot

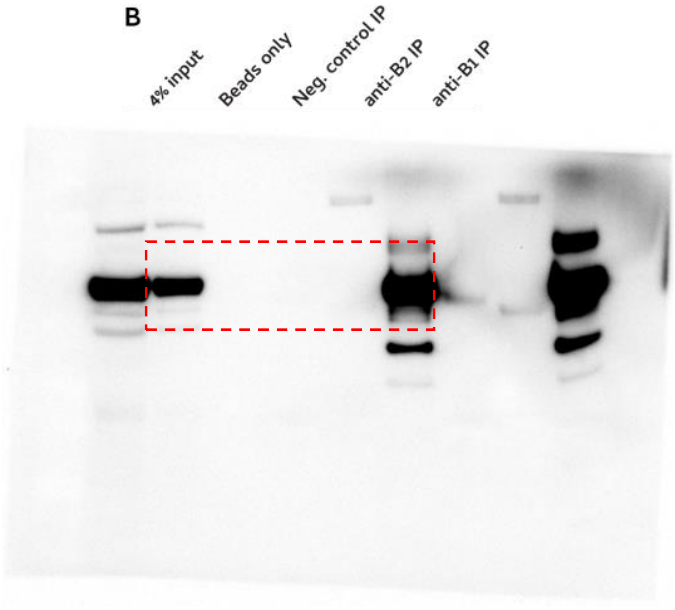

ECL (G:BOX mini imaging system, Syngene, Synoptics)

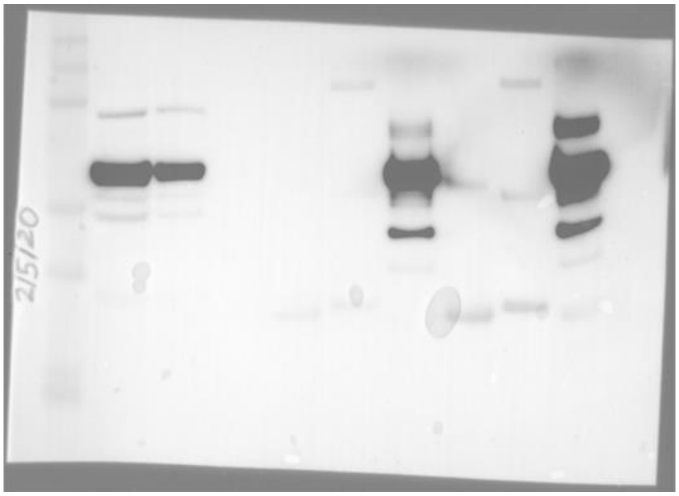

ECL + Visible (G:BOX mini imaging system, Syngene, Synoptics)

Fig. 4A. Anti-Tldc1 western blot

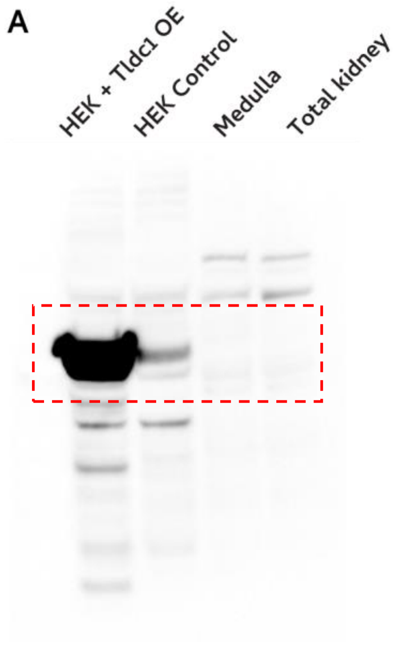

ECL (G:BOX mini imaging system, Syngene, Synoptics)

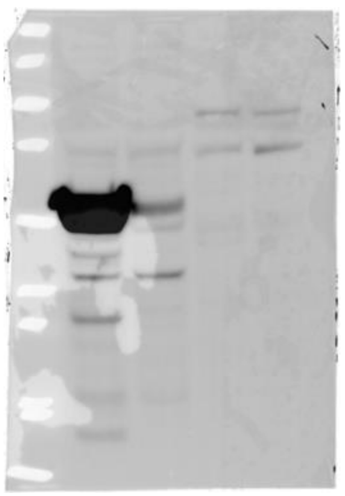

ECL + Visible (G:BOX mini imaging system, Syngene, Synoptics)

Fig. 4A. Anti-β-actin western blot

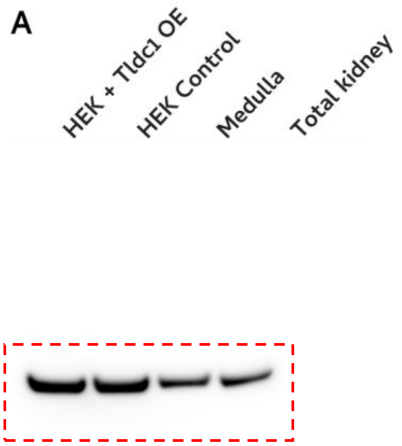

ECL (G:BOX mini imaging system, Syngene, Synoptics)

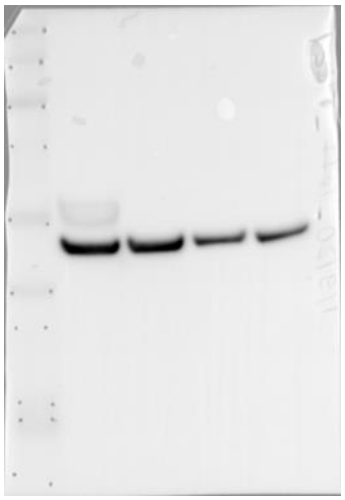

ECL + Visible (G:BOX mini imaging system, Syngene, Synoptics)

Fig. 4B. Anti-Tldc2 western blot

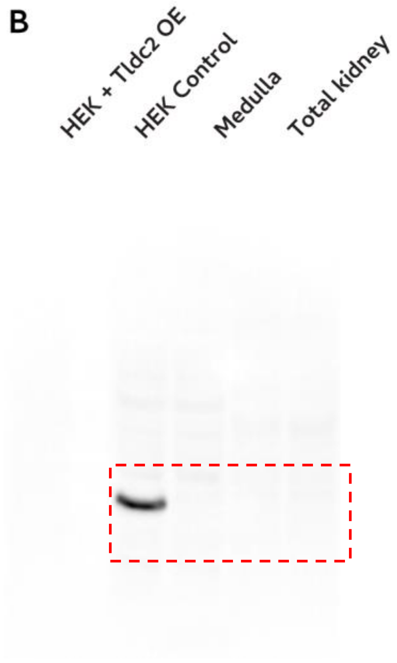

ECL (G:BOX mini imaging system, Syngene, Synoptics)

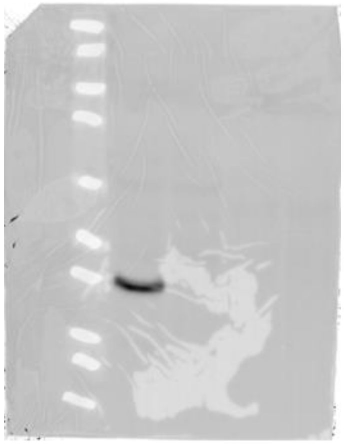

ECL + Visible (G:BOX mini imaging system, Syngene, Synoptics)

Fig. 4B. Anti-β-actin western blot

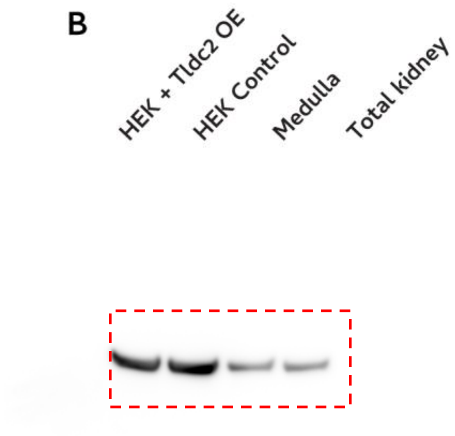

ECL (G:BOX mini imaging system, Syngene, Synoptics)

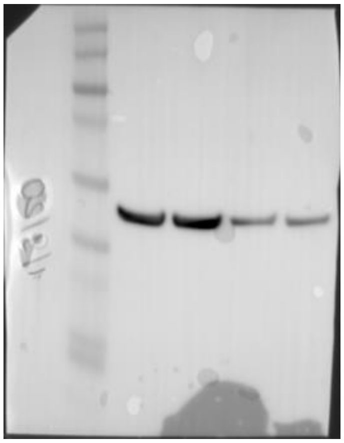

ECL + Visible (G:BOX mini imaging system, Syngene, Synoptics)

Fig. 4C. Anti-Tldc1 western blot

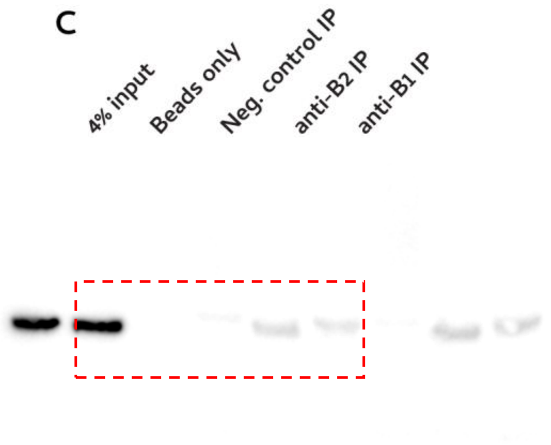

ECL (G:BOX mini imaging system, Syngene, Synoptics)

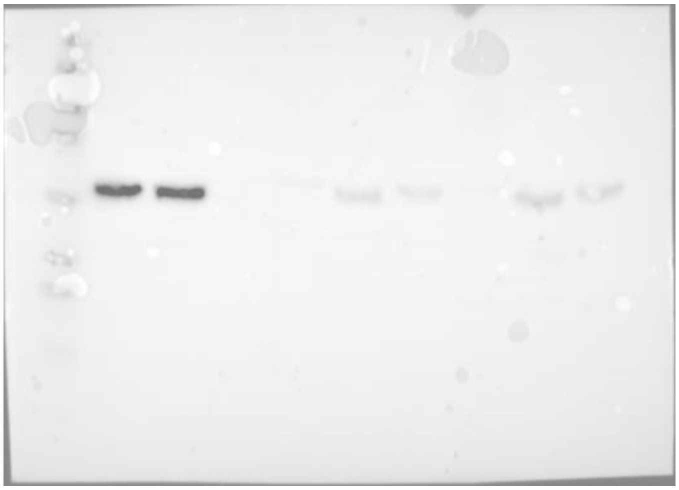

ECL + Visible (G:BOX mini imaging system, Syngene, Synoptics)

Fig. 4C. Anti-B2 western blot

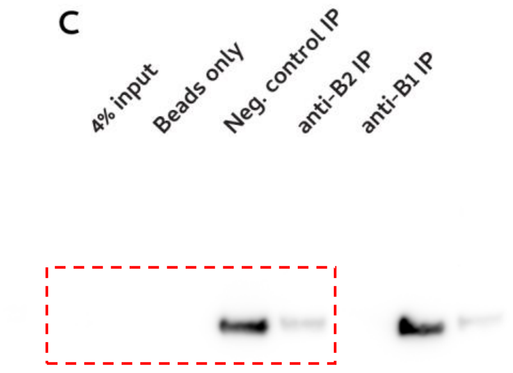

ECL (G:BOX mini imaging system, Syngene, Synoptics)

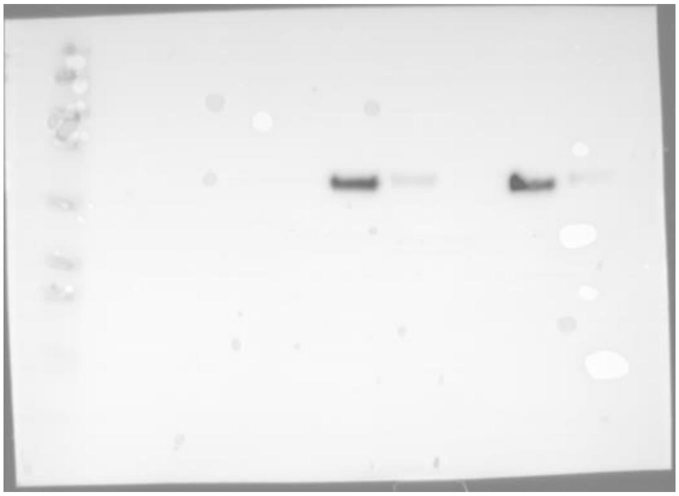

ECL + Visible (G:BOX mini imaging system, Syngene, Synoptics)

Fig. 4C. Anti-B1 western blot

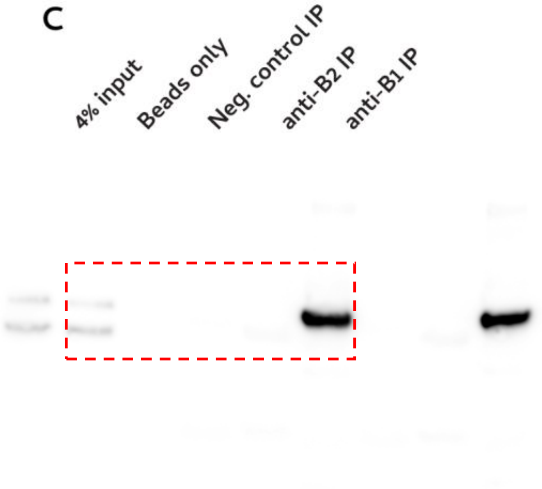

ECL (G:BOX mini imaging system, Syngene, Synoptics)

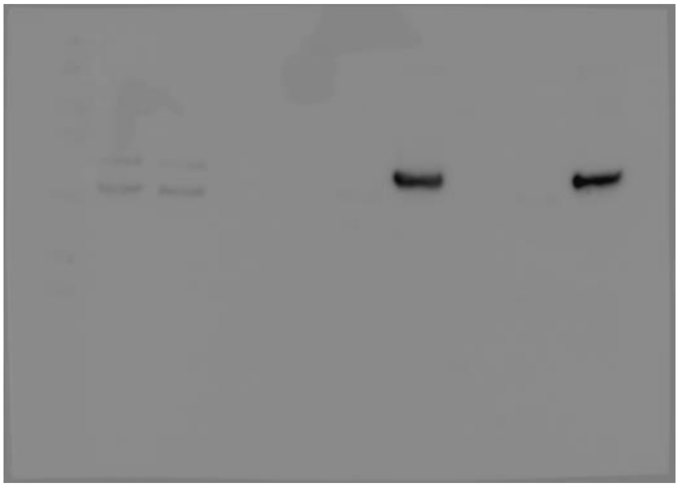

ECL + Visible (G:BOX mini imaging system, Syngene, Synoptics)

Fig. 4D. Anti-Tldc2 (HA) western blot

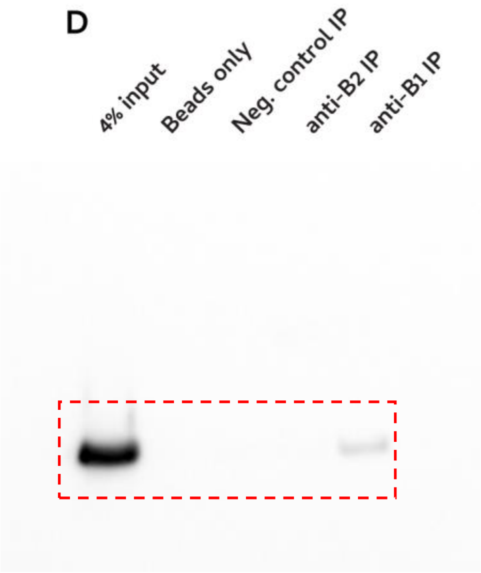

ECL (G:BOX mini imaging system, Syngene, Synoptics)

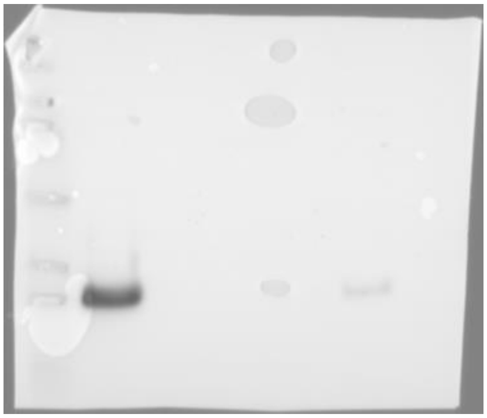

ECL + Visible (G:BOX mini imaging system, Syngene, Synoptics)

Fig. 4D. Anti-B2 western blot

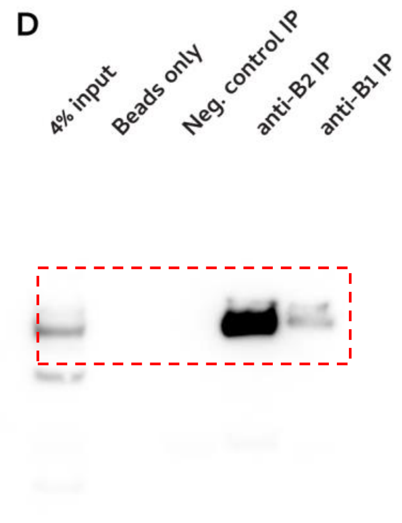

ECL (G:BOX mini imaging system, Syngene, Synoptics)

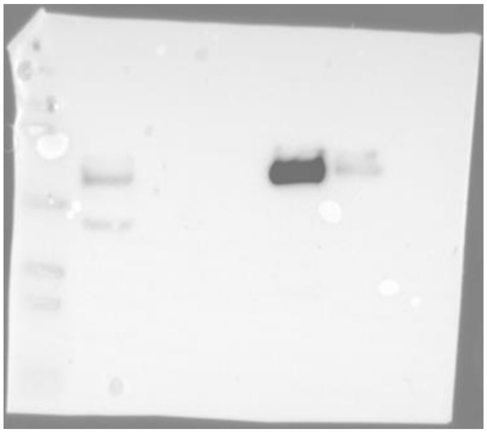

ECL + Visible (G:BOX mini imaging system, Syngene, Synoptics)

Fig. 4D. Anti-B1 western blot

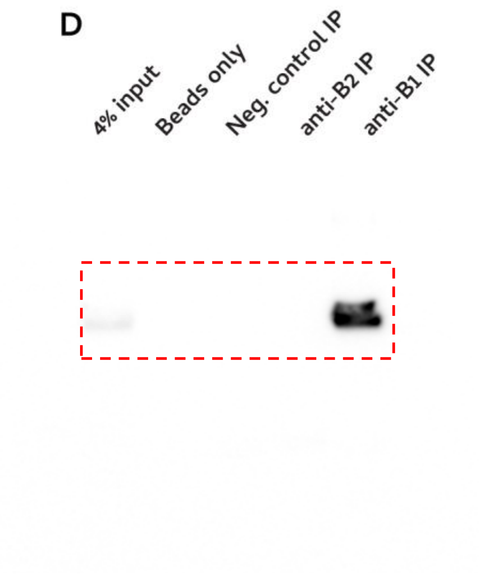

ECL (G:BOX mini imaging system, Syngene, Synoptics)

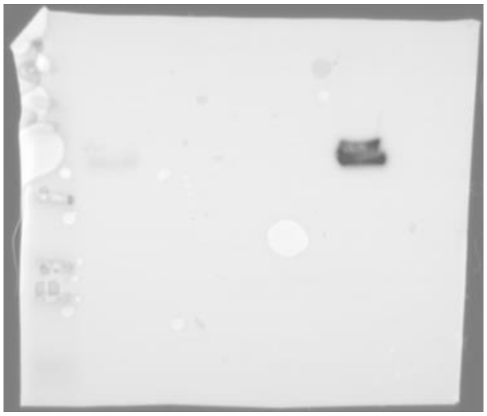

ECL + Visible (G:BOX mini imaging system, Syngene, Synoptics)

Fig. 5B. Anti-B1 western blot

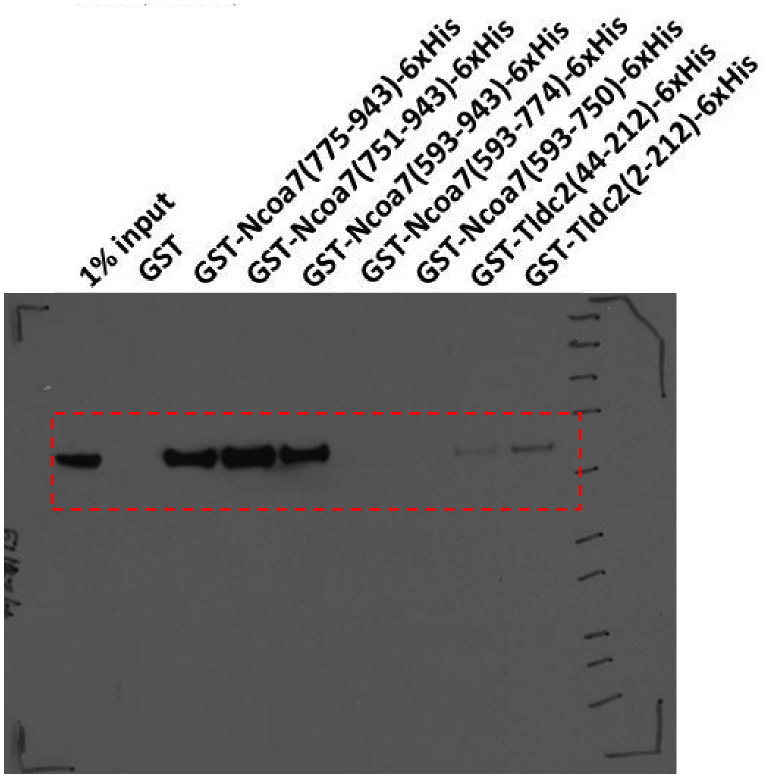

Film, short exposure

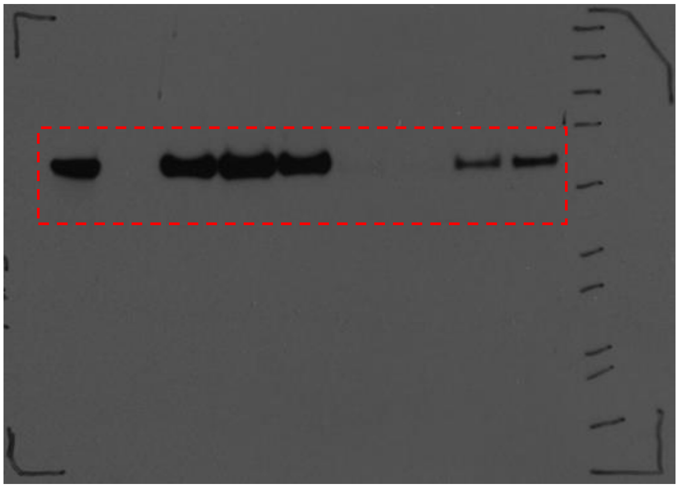

Film, long exposure

Fig. 5B. Anti-GST western blot

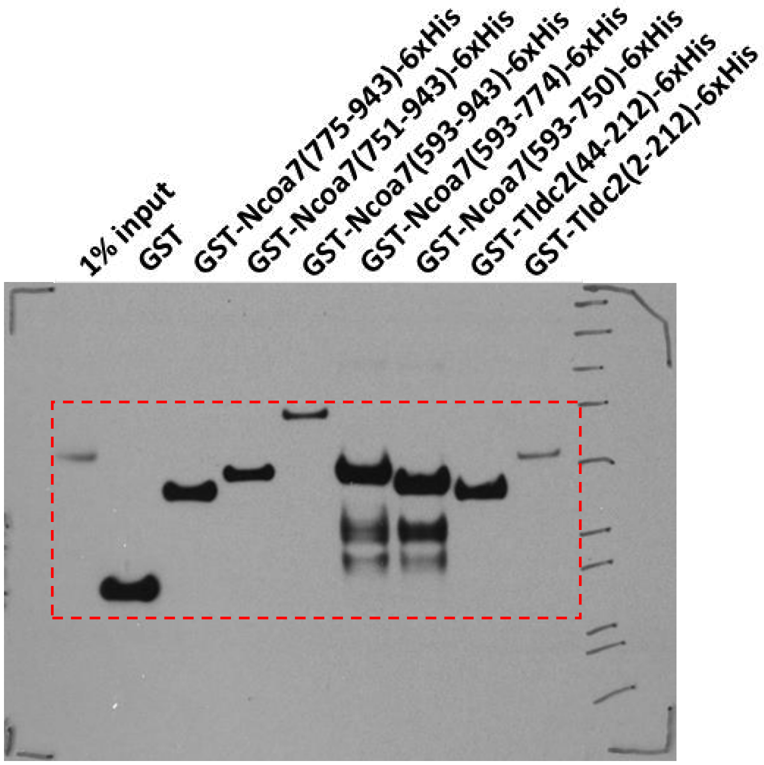

Film

Fig. 6B. Anti-B1 western blot

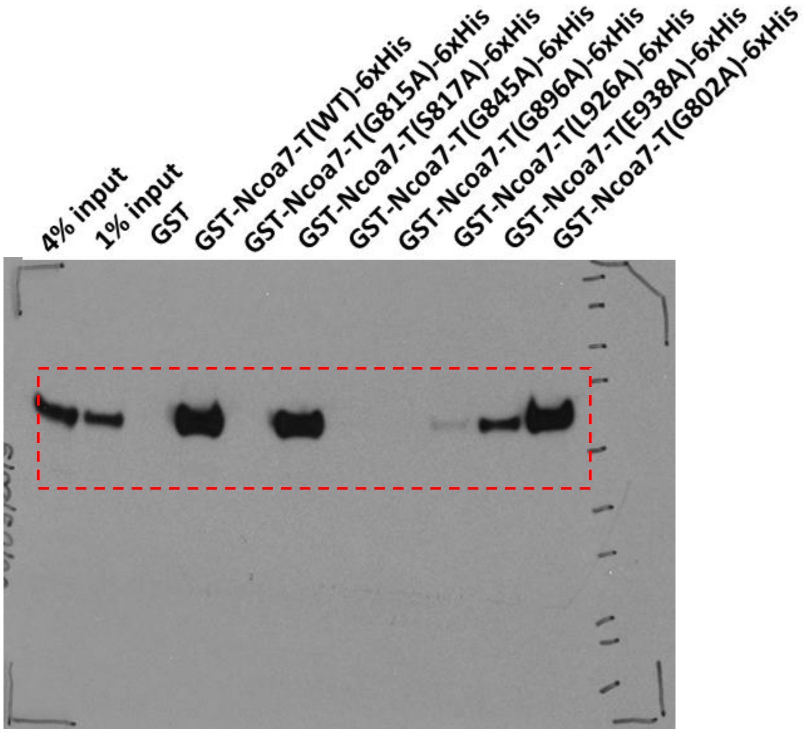

Film

Fig. 6B. Anti-GST western blot

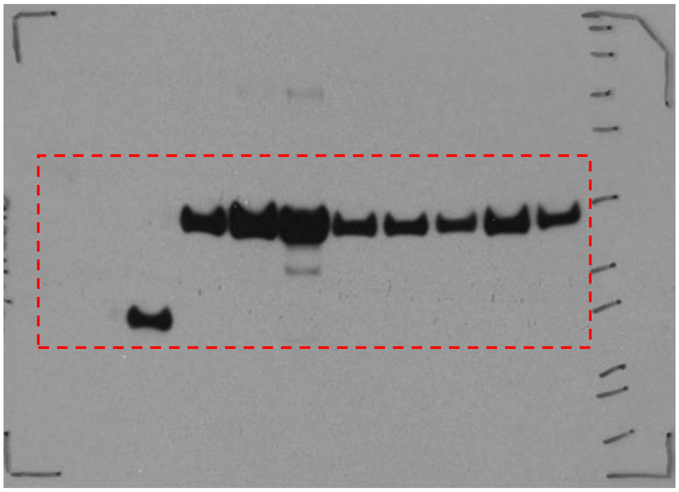

Film

Suppl. Fig. S2. Anti-Tldc1 western blot

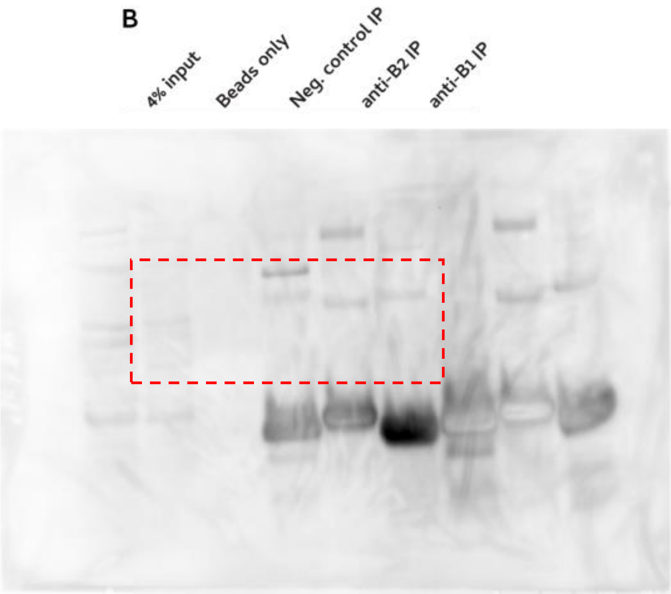

ECL (G:BOX mini imaging system, Syngene, Synoptics)

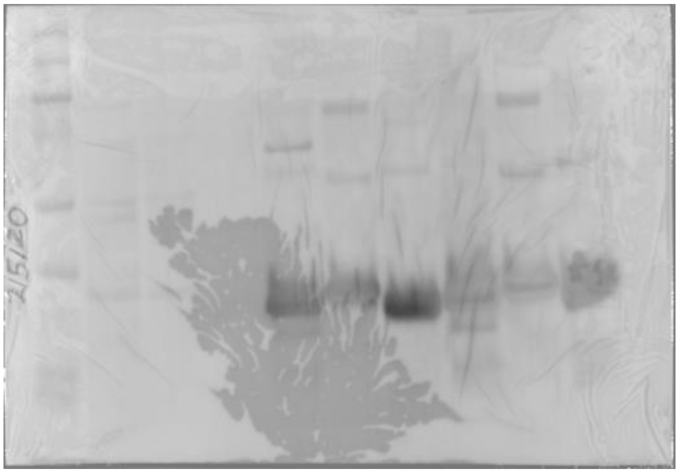

ECL + Visible (G:BOX mini imaging system, Syngene, Synoptics)

Suppl. Fig. S2. Anti-Tldc2 western blot

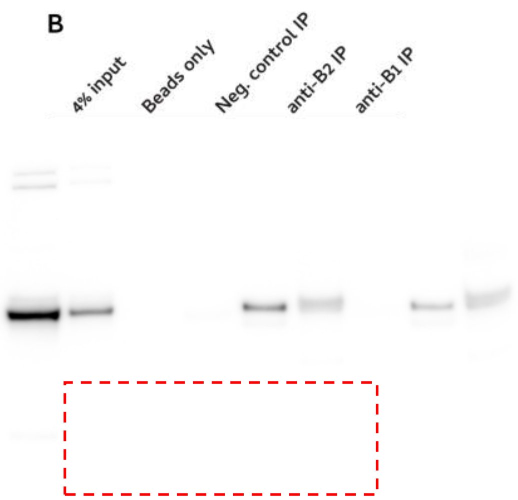

ECL (G:BOX mini imaging system, Syngene, Synoptics)

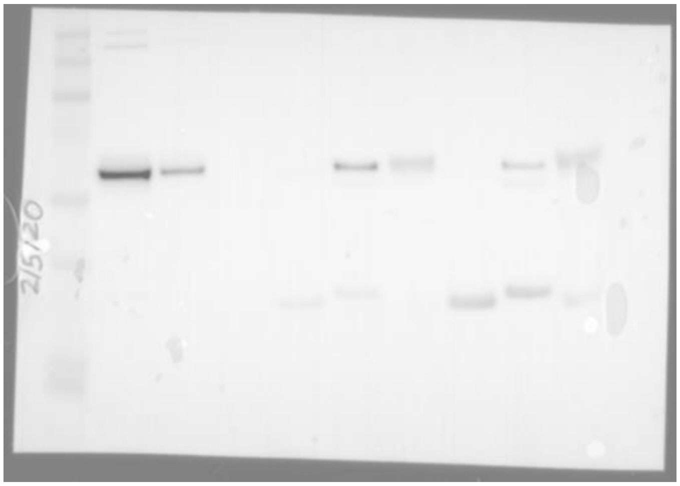

ECL + Visible (G:BOX mini imaging system, Syngene, Synoptics)

Suppl. Fig. S2. Anti-B2 western blot

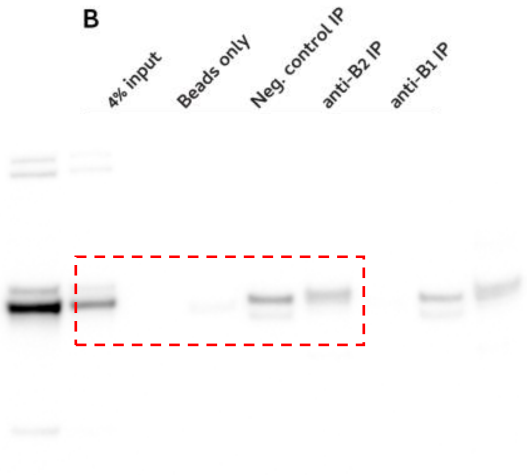

ECL (G:BOX mini imaging system, Syngene, Synoptics)

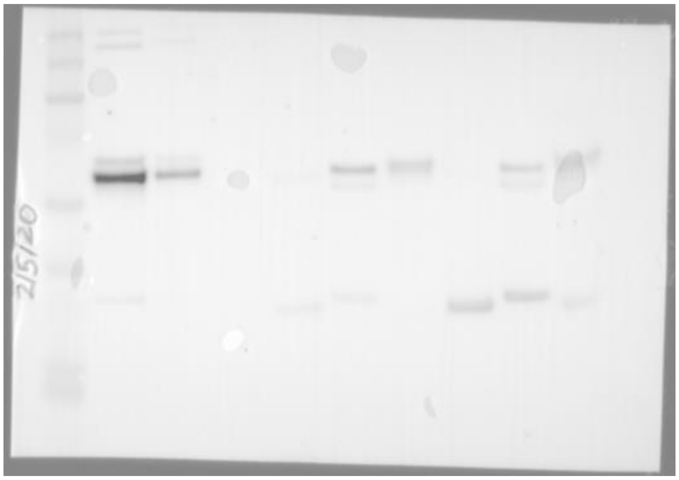

ECL + Visible (G:BOX mini imaging system, Syngene, Synoptics)

Suppl. Fig. S2. Anti-B1 western blot

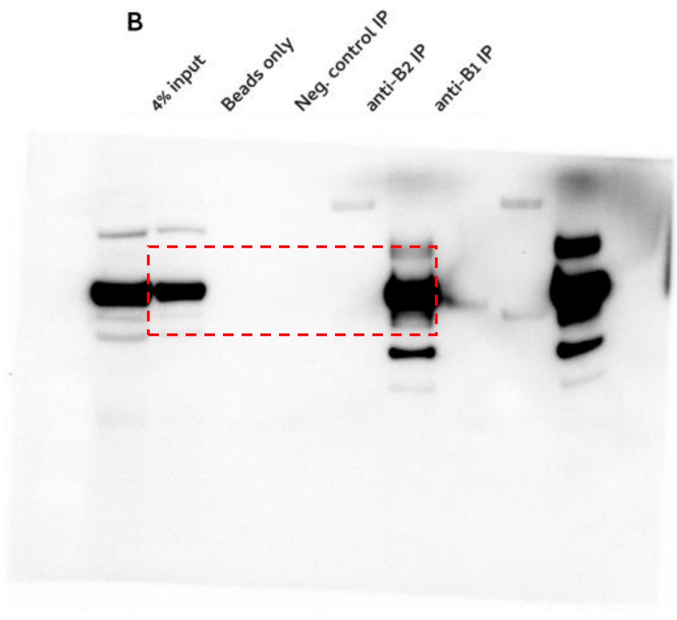

ECL (G:BOX mini imaging system, Syngene, Synoptics)

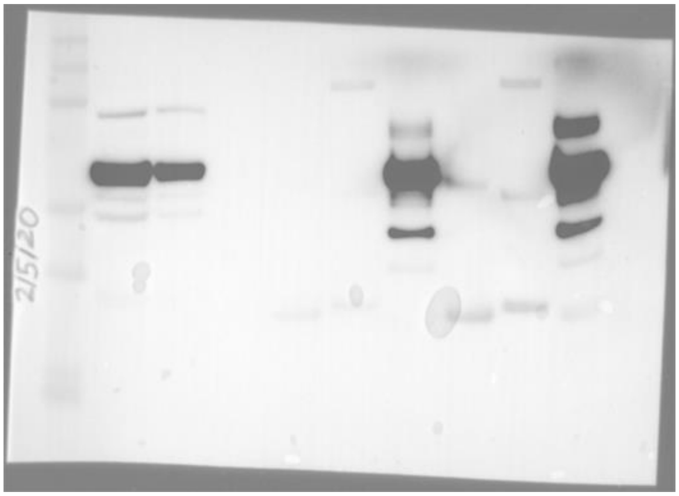

ECL + Visible (G:BOX mini imaging system, Syngene, Synoptics)

Suppl. Fig. S3

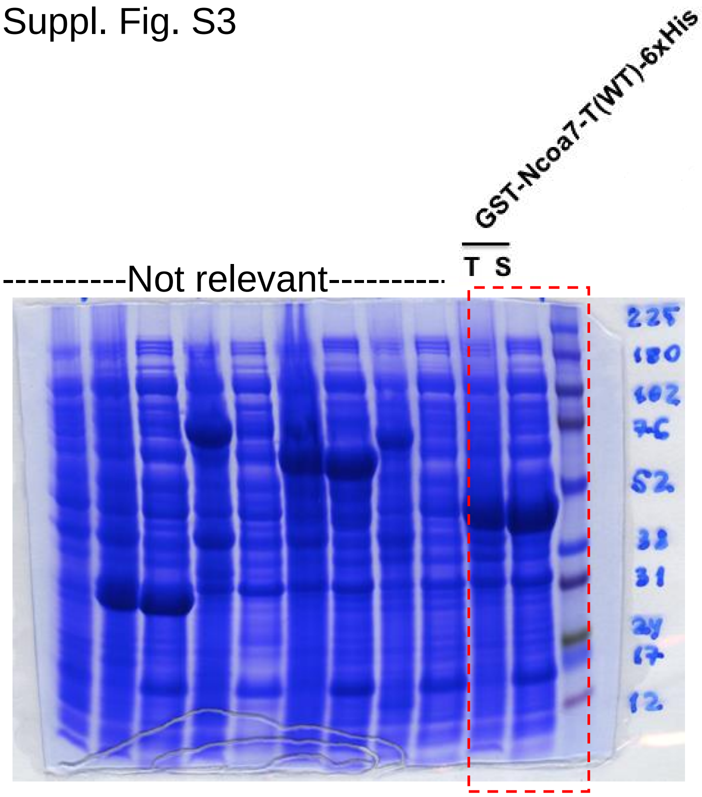

Coomassie Blue R-250 – stained NuPAGE Gel 1

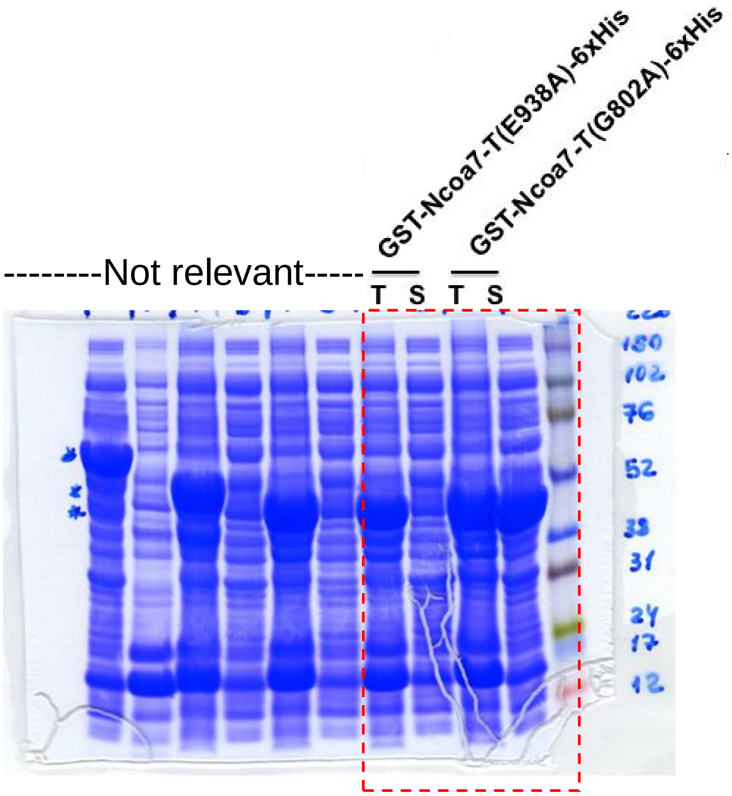

Coomassie Blue R-250 – stained NuPAGE Gel 2

Suppl. Fig. S3, continued

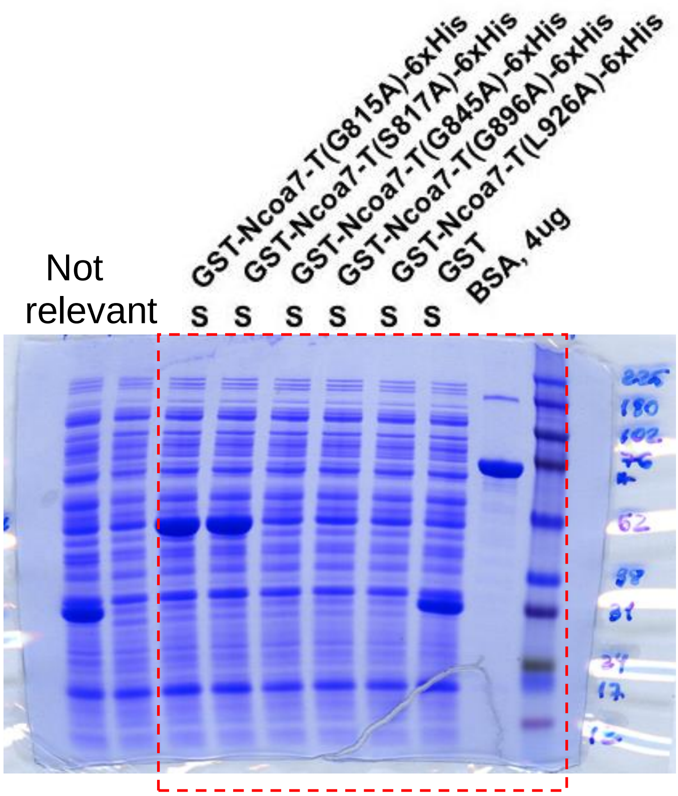

Coomassie Blue R-250 – stained NuPAGE Gel 3
